# Ameliorative Effects of Newly Developed Citrus Hybrid “Mubong” Peel Extract on Experimental Colitis and Gut Microbiota Dysbiosis

**DOI:** 10.1002/fsn3.72098

**Published:** 2026-07-09

**Authors:** Awraris Derbie Assefa, Sang Suk Kim, Seung‐Gab Han, YoSup Park, Jee‐Soo Park

**Affiliations:** ^1^ Citrus Research Center National Institute of Horticultural & Herbal Science, Rural Development Administration Seogwipo Republic of Korea; ^2^ Department of Herbal Crop Research National Institute of Horticultural & Herbal Science, Rural Development Administration Eumseong Republic of Korea; ^3^ Department of Horticultural Science, School of Horticulture and Forest Mokpo National University Muan Republic of Korea

**Keywords:** chemical composition, gut microbiota, “Mubong”, NF‐κB pathway, short‐chain fatty acids, ulcerative colitis

## Abstract

“Mubong,” a newly developed citrus hybrid, is recognized for its unique flavor profile, yet its chemical composition and therapeutic potential remain unexplored. In the present work, we profiled the phytochemical content and antioxidant capacities of “Mubong” flesh and peel, then evaluated the anti‐inflammatory effects of the “Mubong” Peel Extract (MPE) using in vitro (LPS‐stimulated RAW 264.7 cells) and in vivo (DSS‐induced colitis) models, coupled with microbiota analysis. In vitro anti‐inflammatory activity was studied by measuring nitric oxide (NO) production and NF‐κB signaling in RAW‐Blue cells. In vivo, ICR mice were administered MPE (400 mg/kg) orally during DSS‐induced colitis. Disease severity was evaluated through the Disease Activity Index (DAI), colon length, and histological analysis. To characterize microbial and metabolic shifts, we integrated 16S rRNA hypervariable region sequencing with targeted quantification of short‐chain fatty acids (SCFAs). MPE was found to be rich in naringin (2730.66 ± 93.90 mg/100 g), neohesperidin (1493.85 ± 82.67 mg/100 g), and d‐limonene (69.06%). In vitro, MPE (200–400 μg/mL) significantly inhibited NO production and suppressed NF‐κB‐dependent transcriptional activity. In mice, MPE treatment was associated with the attenuation of weight loss, reduction of DAI scores, and the mitigation of colon shortening. These clinical improvements were also coincided with a reduction in pro‐inflammatory cytokines, specifically TNF‐α (~80 to ~52 pg/mL) and IL‐6 (~30 to ~3 pg/mL), and restoration of the SCFAs, propionic acid (~35% to ~80%) and butyric acid (~30% to ~50%). Microbiota analysis revealed that MPE treatment correlated with alterations in the gut landscape, specifically the enrichment of obligate anaerobes such as *Lachnoclostridium* and *Acetatifactor*. Furthermore, these microbial changes paralleled a recovery trend in short‐chain fatty acids (butyrate and propionate) otherwise depleted by DSS. Our findings suggest that MPE exerts anti‐inflammatory activity, likely through the modulation of NF‐κB/AP‐1‐dependent transcriptional activity and preservation of gut microbial homeostasis. These preclinical findings suggest that “Mubong” warrants further investigation as a potential functional food candidate for the management of ulcerative colitis and related inflammatory disorders.

## Graphical abstract

DSS exposure induces severe colitis, causing gut dysbiosis and mucosal inflammation driven by NF‐κB activation and spikes in pathogenic bacteria, TNF‐α, and IL‐6. However, “Mubong” Peel Extract effectively disrupts this inflammatory cascade. By suppressing NF‐κB‐dependent transcriptional activity, the extract mitigates clinical symptoms like colon shortening. This intervention reshapes the gut landscape by enriching beneficial obligate anaerobes, such as *Lachnoclostridium* and *Acetatifactor*. These microbial shifts directly parallel a recovery in short‐chain fatty acids (SCFAs), notably boosting propionate and butyrate levels. Consequently, pro‐inflammatory cytokines drop significantly, restoring gut microbial homeostasis and providing anti‐inflammatory protection.

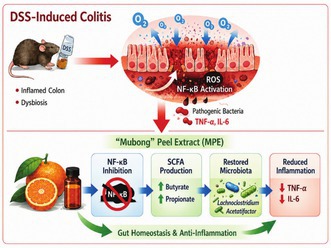

## 1 Introduction

Inflammatory bowel disease (IBD) refers to a group of chronic inflammatory conditions affecting the gastrointestinal tract. Unlike many other digestive conditions, IBD is marked by chronic, relapsing inflammation of the gut, often resulting in severe and debilitating symptoms. Crohn's disease and ulcerative colitis represent the primary clinical manifestations of IBD (Baumgart and Carding 2007; M'Koma 2013). Although the precise etiology of IBD remains elusive, it is widely considered a multifactorial condition. It likely arises from a complex interplay between a patient's genetic susceptibility, external environmental triggers, and a dysfunctional immune system that inappropriately targets the intestinal microbiota (Baumgart and Carding 2007; Loftus 2004). The global prevalence of IBD is on the rise, with the highest rates historically seen in North America, Europe, and Australia. While these regions still have a high number of cases, the most significant increases are now being observed in newly industrialized nations in the Middle East, South America, and Asia (Kaplan and Ng 2017; Kim and Kim 2010; Loftus 2004). The disease is most commonly diagnosed in young adults, with slight variations between genders (M'Koma 2013). According to a study by the Global Burden of Disease (GBD), IBD affected an estimated 6.8 million people worldwide as of 2017 (Liu, Li, et al. 2024). Projections indicate that newly industrialized countries will transition from a low‐prevalence, high‐incidence phase to a compounding prevalence over the next two decades (Kaplan 2025).

Treatments for IBD primarily aim to temporarily reduce inflammation using a range of medications and, occasionally, surgery; however, they do not offer a complete cure. Medications include 5‐aminosalicylates for mild cases (Domènech 2006), with side effects like headaches and nausea (Park and Cheon 2022; Xie et al. 2019), corticosteroids for short‐term flare‐ups, which can cause significant side effects such as headache, acne, electrolyte imbalance, hypertension, hyperglycemia, susceptibility to infection, aseptic joint necrosis, osteoporosis, and adrenal insufficiency; and immunomodulators and biologics for long‐term management, which increase the risk of infections (Park and Cheon 2022). For severe cases unresponsive to medication, surgery may be necessary, but it also carries risks like infection and bleeding (Rönnblom et al. 2021). Hence, developing food‐based products is a promising strategy for managing IBD. Functional foods can serve as a complementary alternative to conventional medicine, utilizing natural bioactive compounds to reduce inflammation and support gut health. This approach is highly appealing to patients seeking to manage their condition with fewer side effects. There is substantial evidence from in vitro models, animal studies, and preliminary human trials that various plant‐based compounds can help alleviate intestinal inflammation, primarily owing to their capacity to regulate key inflammatory pathways within the gut. Some prominent examples include curcumin in turmeric (Balaji et al. 2025), gingerols and shogaols in ginger (Zhang et al. 2017), epigallocatechin gallate in green tea (Du et al. 2019), resveratrols in grapes and berries (Alrafas et al. 2019), and flavonoids in citrus fruits (Stevens et al. 2022).

Colitis induced by Dextran Sulfate Sodium (DSS) is an established murine model that serves as a faithful representation of the inflammatory profile observed in human ulcerative colitis patients (Chassaing et al. 2014; Eichele and Kharbanda 2017; Yang and Merlin 2024). In this model, DSS is supplied in the animals' drinking water, damages the colon's epithelial barrier and allows bacteria to penetrate the underlying tissue, triggering a robust inflammatory response (Chassaing et al. 2014). The model is validated for IBD research as it replicates the clinical symptoms seen in humans, such as weight loss, diarrhea, rectal bleeding, and histopathological features including mucosal damage and immune cell infiltration (Eichele and Kharbanda 2017; Melgar et al. 2008; Sha et al. 2013). Additionally, this model is characterized by an inflammatory cascade involving the same molecular mediators and signaling networks, such as NF‐κB, that underpin the clinical progression of human IBD. Although the DSS model is an acute, chemically induced model and does not fully capture the chronic, genetic, and environmental complexity of human disease, its reproducibility and ability to replicate core inflammatory mechanisms make it an indispensable tool for initial drug screening and for studying the fundamental basis of intestinal inflammation (Chandra et al. 2025; Eichele and Kharbanda 2017; Yang and Merlin 2024).

Citrus fruits, long consumed for thousands of years and valued for their medicinal properties, contain a wide range of bioactive compounds that confer health benefits such as anti‐inflammatory and antioxidant effects. The primary components responsible for these effects are flavonoids, especially flavanones like hesperidin and naringin, polymethoxyflavonoids (PMFs) and their hydroxylated derivatives, and limonoids, which are abundantly found in both the peel and pulp of citrus fruits (Fontana et al. 2023; Lv et al. 2015; Saini et al. 2022; Wang et al. 2018; Zou et al. 2016). These compounds work together by inhibiting key inflammatory signaling pathways like NF‐κB and MAPK, thereby suppressing the production of pro‐inflammatory molecules and enzymes such as TNF‐α, IL‐1β, COX‐2, and iNOS. These bioactive constituents exhibit significant antioxidant activity, which may play role in limiting the oxidative stress known to exacerbate chronic inflammatory conditions. Extracts from citrus fruits have also shown promise as anti‐inflammatory agents (Lee et al. 2013; Malleshappa et al. 2018).

A new citrus cultivar “Mubong” was developed at the Citrus Research Center in Seogwipo, Jeju Island, through a cross between “Shiranui” [(*C. unshiu* Marc × *C. sinensis* Osb.) × “Nagano No. 3” Ponkan ( *C. reticulata* Blanco)] and “Hassaku” (*C. hassaku* Hort.). Officially registered in 2020, “Mubong” produces highly marketable, seedless fruits averaging 300 g with high yields. Although “Mubong” has recently gained attention as a commercially valuable citrus cultivar, its bioactive metabolite profile and anti‐inflammatory potential have not yet been systematically investigated. We have therefore undertaken an investigation to characterize these properties. While “Mubong” is a newly developed hybrid, our study is the first to systematically evaluate its metabolite profile and its effect on gut health and inflammation. Furthermore, we assessed the impact of “Mubong” extract intake on the gut microbiota profile, an index of colitis symptoms known to be intrinsically related to the IBD inflammatory response. Based on these uncharacterized properties, we posited that “Mubong” citrus peels would alleviate the symptoms of acute colitis by modulating intestinal inflammation and gut permeability in a murine model.

## 2 Materials and Methods

### 2.1 Biochemical Analysis

#### 2.1.1 Fruit Sample Preparation

Fruit samples were obtained from the experimental field of the Citrus Research Center, Seogwipo, Korea. Fruits were harvested at commercial maturity based on visual assessment of uniform size and external color. The surface of the fruits was gently cleaned using distilled water and dehydrated. The peel and flesh were manually separated. To determine free sugar and organic acid composition, fresh juice of the pulp was used. To study the antioxidant activities, flavonoids and anti‐inflammatory properties, samples were dried at 55°C, powdered and extracted using 70% ethanol at a 20:1 (v/w) solvent‐to‐sample ratio. To optimize recovery, the extracts were sonicated for 60 min at 37°C to facilitate cellular disruption, followed by high‐speed centrifugation (10,000 *g*, 10 min, 4°C) to remove non‐soluble remnants. The supernatant was purified through fine‐pore filtration to ensure quality and subsequently concentrated via rotary evaporation at 45°C under reduced pressure, allowing for efficient solvent removal while safeguarding the extract's thermolabile constituents from thermal degradation. The extraction process was carried out using a standardized method previously optimized in our lab and validated across various citrus cultivars. This extraction framework is standardized against the major citrus flavonoids: rutin, narirutin, naringin, hesperidin, neohesperidin, quercetin, naringenin, hesperetin, nobiletin, and tangeretin. The robustness and reproducibility of this platform method were confirmed by evaluating independent extraction batches across cultivars, demonstrating strict batch‐to‐batch consistency for the target marker compounds. For the present study, “Mubong” was processed strictly under this validated protocol, ensuring a highly reproducible phytochemical matrix for the downstream biological evaluations.

#### 2.1.2 Analysis of Physicochemical Characters

The total soluble solids (TSS) content was measured using a digital refractometer. Freshly extracted juice was subjected to centrifugation at 10,000 *g* (15 min, 4°C), and subsequently the supernatant was collected for TSS analysis. Titratable acidity (TA) was determined by titrating a diluted supernatant (2 mL in 8 mL distilled water) with 0.1 N NaOH to an end point of pH of 8.1, and results were expressed as citric acid equivalents. Free sugars and citric acid were quantified via HPLC after tenfold juice dilution and 0.45 μm filtration. Sugars were separated on Agilent ZORBAX NH_2_ column (4.6 × 250 mm, 5 μm) using isocratic 75% acetonitrile (1 mL/min, 40°C) coupled with a Shimadzu refractive index detector (RID‐20A). Citric acid was analyzed on a Shimadzu Shim‐Pak GIS C_18_ column (4.6 × 250 mm, 5 μm) at 30°C using a 10 mM sodium phosphate (pH 2.6)/acetonitrile gradient (flow rate, 1 mL/min) and UV detection at 226 nm. Concentrations were determined using external calibration curves.

#### 2.1.3 Antioxidant Activity

The antioxidant potentials were evaluated using three well‐established assays: 2,2‐diphenyl‐1‐picrylhydrazyl (DPPH), 2,2′‐azino‐bis‐3‐ethylbenzthiazoline‐6‐sulphonic acid (ABTS), and ferric reducing antioxidant power (FRAP).

The DPPH radical scavenging activity was evaluated based on the method of Blois (1958). Briefly, a 0.2 mM DPPH solution was mixed with various concentrations of the samples. After a 10‐min incubation at room temperature, the absorbance was measured at 517 nm, and the results were reported as the IC_50_ value, representing the concentration required for 50% radical scavenging. The ABTS radical scavenging activity was determined using a modified decolorization assay as described by Re et al. (1999). An ABTS radical cation was generated by reacting a 7.4 mM ABTS stock solution with 2.6 mM potassium persulfate, followed by a 15‐h dark incubation. The solution's absorbance was adjusted to 0.70 ± 0.02 at 734 nm. For the assay, 20 μL of the extract was added to 180 μL of the ABTS solution, incubated for 15 min, and the absorbance was measured at 734 nm. The scavenging activity was then expressed as the percentage of absorbance reduction relative to the control. These methods collectively provided a comprehensive assessment of the samples' antioxidant capacity. The DPPH and ABTS antioxidant activities were calculated using the following equation:

$$\text{DPPH}/\text{ABTS inhibition}\;\left(\%\right)=\left(\frac{A_{\text{Control}}-A_{\text{Sample}}}{A_{\text{Control}}}\right)\times 100$$

where $A_{\text{Control}}$ = Absorbance of DPPH/ABTS solution without sample; where $A_{\text{Sample}}$ = Absorbance of mixture of DPPH/ABTS and sample solution. The absorbance was measured using a SpectraMax M3 Multi‐Mode Microplate Reader (Molecular Devices, Sunnyvale, CA, USA). Ascorbic acid was used as a control and results were reported as ascorbic acid equivalents.

The FRAP of the “Mubong” extract was determined using a method adapted from Benzie and Strain (1996). The FRAP reagent was prepared immediately before use by mixing a solution of 300 mM acetate buffer (pH 3.6), 10 mM TPTZ (in 40 mM HCl), and 20 mM FeCl_3_·6H_2_O in a ratio of 10:1:1 (v/v/v). The test solution was created by mixing 3 mL of the FRAP reagent, 0.3 mL of water, and 0.1 mL of the test sample or standard solution. After a 30‐min incubation at 37°C, the absorbance was measured at 593 nm using a SpectraMax M3 Multi‐Mode Microplate Reader. The results were reported as ascorbic acid equivalents.

#### 2.1.4 Total Phenolic (TP) and Total Flavonoid (TF) Content

The TP content was measured using a modified Folin–Ciocalteu method as described in a previous study (Assefa et al. 2017). Briefly, 100 μL of the extract stock solution was combined with 100 μL of Folin–Ciocalteu reagent and 900 μL of water. After a 5‐min incubation at room temperature, 200 μL of a 7% (w/v) sodium carbonate solution was added. The reaction was allowed to proceed for an additional 60 min. The absorbance was then measured at 720 nm using the SpectraMax M3 Multi‐Mode Microplate Reader. All measurements were performed in triplicate, and the results were expressed as gallic acid equivalents.

The TF content was determined using a colorimetric aluminum chloride method as reported in a previous study (Assefa and Keum 2017). An appropriately diluted 0.5 mL sample solution was mixed with 1.5 mL of ethanol, 0.1 mL of a 10% aluminum chloride solution, 0.1 mL of 1 M potassium acetate, and 2.8 mL of deionized water. The mixture was incubated at room temperature for 30 min. The absorbance was subsequently measured at 415 nm using SpectraMax M3 Multi‐Mode Microplate Reader. All measurements were performed in triplicate, and the results were reported as quercetin equivalents.

#### 2.1.5 Quantification of Major Flavonoids

The freeze dried extracts were reconstituted in 70% ethanol, filtered through a 0.22 μm PVDF filter, and analyzed using a Waters e2695 HPLC system equipped with a Waters 2489 UV/Visible Detector. Chromatographic separation was performed on a YMC‐Triart C18 column (250 × 4.6 mm, 5 μm) with a flow rate of 1 mL/min. An isocratic elution was used, with solvent compositions optimized for different flavonoid types: Glycosylated flavonoids were separated using a 2:8 ratio of acetonitrile (ACN) to 20 mM phosphoric acid, and a 6:4 ratio for the less polar polymethoxylated flavonoids. The column temperature was maintained at 45°C, and flavonoids were monitored at 280 nm. Identification was based on a comparison of retention times and characteristic spectra against known standards. Quantification was performed using an external standard method, with calibration curves constructed from each standard at concentrations from 15.625 to 1000 μg/mL.

#### 2.1.6 Analysis of Volatile Organic Compounds (VOCs)

The volatile metabolite profile of the fruit peel was determined using headspace solid‐phase microextraction (HS‐SPME) coupled with gas chromatography–mass spectrometry (GC–MS) according to previously established methods (Kim et al. 2024), with minor modifications. Fresh samples were transferred into headspace‐SPME vials containing 6 mL of distilled water. The vials were sealed and equilibrated at 70°C for 15 min to facilitate the release of volatile compounds into the headspace. Extraction was performed using a 2 cm SPME fiber (50/30 μm, DVB/CAR/PDMS Stableflex; Sigma–Aldrich, St. Louis, MO, USA) exposed to the headspace at 70°C for an additional 15 min. Following extraction, the SPME fiber was inserted into the injection port of an Agilent 7890A GC system (Agilent Technologies, Santa Clara, CA, USA) and desorbed at 200°C for 5 min in splitless mode. Chromatographic separation was achieved using a DB‐WAX capillary column (30 m × 0.25 mm i.d., 0.25 μm film thickness; Agilent Technologies). The oven temperature program was initiated at 40°C (held for 3 min), increased to 90°C at a rate of 4°C/min, further increased to 210°C at 19°C/min, and finally held at 210°C for 9 min. The eluting compounds were detected using an Agilent 5975C Inert XL mass selective detector (MSD). Ionization was performed via electron impact (EI) at 70 eV. The temperatures for the ion source, transfer line, and quadrupole were maintained at 200°C, 250°C, and 150°C, respectively. Mass spectra were acquired in full scan mode over a mass‐to‐charge ratio (*m*/*z*) range of 45–550.

### 2.2 In Vitro Study

#### 2.2.1 Cell Culture and Viability

RAW 264.7 murine macrophages were cultured in Dulbecco's modified Eagle's minimal essential medium (DMEM; Gibco Inc., NY, USA) containing 10% heat‐inactivated fetal bovine serum (FBS; Gibco Inc.) and penicillin/streptomycin (P/S, Gibco Inc.) at 37°C, 5% CO_2_ and 95% humidity. Cell viability was determined using a modified MTT [3‐(4,5‐dimethylthiazol‐2‐yl)‐2,5‐diphenyltetrazolium bromide] assay. RAW264.7 cells were placed (1 × 10^4^ cells/well) into 96‐well culture plate and incubated with 0–400 μg/mL of “Mubong” peel extracts (MPEs) for 1 h and incubated 12 h with 1 μg/mL of LPS. After treatment, 400 μL MTT reagent (0.2 mg/mL) was added to each well and incubated for 4 h at 37°C. The supernatant was then discarded, and the resulting formazan crystals were dissolved in 800 μL of dimethyl sulfoxide (DMSO). Absorbance was measured at 540 nm using microplate reader spectrometer (Sunrise microplate reader, Tecan, Swiss). Cell viability was calculated relative to untreated control cells and expressed as mean ± SD from three independent experiments.

$$\text{Cell viability}\;\left(\%\right)=100\times \frac{A_{t}}{A_{C}}$$

where $A_{t}$ is absorbance of MPE treated cells; $A_{c}$ is absorbance of control.

#### 2.2.2 Nitric Oxide (NO) Production

RAW 264.7 cells were placed (3 × 10^4^ cells/well) into 96‐well culture plate and treated with MPE at concentrations of 0–400 μg/mL for 1 h. Simultaneously, cells were stimulated with 1 μg/mL of LPS and incubated for 24 h at 37°C. After incubation, the culture media were collected from each well to assess nitrite levels using the Griess reagent, composed of 1% sulfanilamide, 0.1% N‐(1‐naphthyl)ethylenediamine, and 2.5% phosphoric acid. Equal volumes of culture medium (100 μL) and Griess reagent (100 μL) were mixed and incubated at room temperature (18°C–25°C) for 15 min. Absorbance was measured at 540 nm using a microplate reader spectrometer (Sunrise microplate reader). Results are presented as the mean ± SD from three independent experiments. Nitric oxide production was calculated as:

$$\mathrm{NO}\;\text{production}\;\left(\%\right)=100\times \frac{A_{t}}{A_{\mathrm{LPS}}}$$

where $A_{t}$ is absorbance of MPE treated cells; $A_{\mathrm{LPS}}$ is absorbance of LPS treated cells.

#### 2.2.3 NF‐κB Activation

The activation of the NF‐κB signaling pathway was evaluated using RAW‐Blue cells, which are derived from RAW 264.7 macrophages. These cells are engineered to express secreted embryonic alkaline phosphatase (SEAP) under the control of a promoter inducible by NF‐κB and AP‐1. RAW‐Blue cells were seeded in 96‐well plates at a density of 3 × 10^4^ cells/well. Following an initial stabilization period, the cells were pre‐treated with various concentrations of MPE (0–400 μg/mL) for 1 h. To stimulate the inflammatory response, the cells were subsequently challenged with 1 μg/mL of lipopolysaccharide (LPS) and incubated for 12 h at 37°C in a 5% CO_2_ atmosphere. To quantify the induced SEAP activity, 20 μL of the cell culture supernatant was collected and transferred to a new 96‐well plate containing 180 μL of QUANTI‐Blue detection medium. The mixture was incubated at 37°C for 1 h to allow for color development. The enzymatic activity, reflecting the degree of NFκB activation, was determined by measuring the absorbance at 620 nm using a microplate reader. The absorbance of vehicle‐treated wells (LPS‐only) was used as a reference to determine the inhibitory effects of the samples. Results are presented as the mean ± SD from three independent experiments.

$$\text{NFκB activation}\;\left(\%\right)=100\times \frac{A_{t}}{A_{\mathrm{LPS}}}$$

where $A_{t}$ is absorbance of MPE treated cells; $A_{\mathrm{LPS}}$ is absorbance of LPS treated cells.

### 2.3 Animal Study and Experimental Design

**FIGURE 1:**
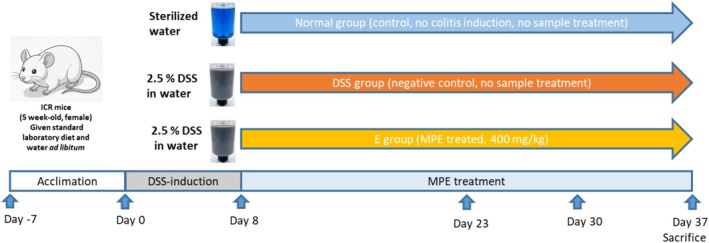
Schematic diagram of administration timeline and dosage.

ICR mouse (Female, 5 weeks old) were purchased from Orient Bio Inc. (Gapyeong, Korea). Upon arrival, the animals were housed under specific pathogen‐free conditions with a controlled temperature of 24°C ± 1°C, a relative humidity of 50%–55%, and a 12/12 h light–dark cycle. All mice were provided with a standard laboratory diet and water ad libitum and were allowed to acclimate to the environment for 7 days prior to the commencement of the study. Following acclimation, the mice were randomly assigned to three experimental groups (*n* = 6 per group): Normal group (control, no colitis induction); DSS group (negative control, DSS‐induced colitis, no sample treatment); and E group (MPE 400 mg/kg with colitis induction). The sample size (*n* = 6 per group) was determined based on previous literature utilizing the DSS‐induced colitis model (Chassaing et al. 2014; Su et al. 2011; Urushima et al. 2015) and aligned with institutional guidelines to minimize animal use (the Reduction principle) while maintaining adequate statistical power to detect biologically meaningful differences. The oral administration dose of the extract (400 mg/kg) was selected based on a preliminary study conducted in our laboratory, which demonstrated optimal efficacy and a favorable safety profile in experimental models. Following an initial acclimatization period, colitis was induced by supplementing the drinking water with 2.5% (w/v) DSS from day 0 to day 7. For the remainder of the experimental period (days 8–37), the E group received daily oral administration of MPE. The DSS‐induced colitis model was established according to the timeline and concentrations detailed in Figure 1.

To evaluate the clinical severity of colitis, the disease activity index (DAI) was assessed and recorded on days 8, 23, 30, and 37 of the experimental period. The DAI was calculated as the cumulative score of three primary clinical parameters: body weight loss, stool consistency, and the presence of gross blood. The specific scoring criteria utilized for the DAI are detailed in Table 1. At the conclusion of the experimental period, mice were subjected to a 12‐h fast. The animals were then humanely euthanized via CO_2_ inhalation in accordance with established ethical guidelines. All experimental protocols were conducted in strict accordance with the institutional and national regulatory frameworks governing the ethical care and use of laboratory animals.

**TABLE 1:** Scoring of disease activity index (DAI).

Score	Weight loss (%)	Stool consistency	Occult/gross bleeding
0	None	Normal	Normal
1	1%–5%	Slightly loose stool	Anal edema
2	6%–10%	Loose stools	Occult bleeding
3	10%–15%	Watery stool	Intermediate bleeding
4	> 15%	Diarrhea	Gross bleeding

#### 2.3.1 Histopathological Assessment

Terminal procedures occurred on Day 37, including euthanasia and colon excision. Representative images were captured with the tissues positioned against a standardized metric background to provide a clear indication of scale before the tissue was fixed in 4% paraformaldehyde. Tissue sections (3 μm) were prepared using a microtome (Finesse ME Microtome, Thermo Shandon, UK). Slides were stained with H&E and immunostained for F4/80 (Abcam, Cambridge, MA, USA). Pathological assessment followed established criteria, comparing necrosis, inflammation, and mucosal damage based on the International Harmonization of Nomenclature and Diagnostic Criteria. Histopathological assessment was performed by an independent researcher who was blinded to the treatment groups.

#### 2.3.2 Analysis of Short‐Chain Fatty Acids (SCFAs) in Feces

Fecal SCFA concentrations were determined via gas chromatography–mass spectrometry (GC–MS) following a liquid–liquid extraction procedure. Briefly, 100 mg of fecal matter was homogenized in 800 μL of 0.1 M sulfuric acid (H_2_SO_4_) and vortexed thoroughly to facilitate the extraction of organic acids. The suspension was subjected to centrifugation at 12,000 *g* for 10 min (4°C) to facilitate phase separation. SCFAs were isolated by mixing the collected supernatant with 800 μL of diethyl ether. The mixture underwent an additional extraction step involving vortexing and centrifugation (12,000 *g*, 10 min), after which the upper organic layer was recovered for GC–MS quantification. The analysis was performed using an Agilent 6890 Gas Chromatograph coupled with an Agilent 5973 Mass Selective Detector (Agilent Technologies, Santa Clara, CA, USA). Compounds were separated on a DB‐5 ms capillary column (30 m × 0.25 mm i.d., 0.25 μm film thickness; Agilent Technologies). Chromatographic separation was achieved using helium as the carrier gas at a flow rate of 0.8 mL/min and a split ratio of 1:10. The thermal gradient commenced at 50°C, ascending at 3°C/min until a terminal temperature of 80°C was reached. Consistent temperatures were established for the injector (250°C) and detector (270°C). Identification of SCFAs was performed through spectral matching against the NIST database, while quantification was executed using standard external calibration curves.

#### 2.3.3 Quantification of Inflammatory Cytokines

Blood samples were collected from the mice's inferior vena cava and centrifuged at 2000 *g* for 20 min to separate the serum. The serum was then stored in a sterilized tube at −60°C until analysis. Pro‐inflammatory cytokine levels, specifically TNF‐α and IL‐6, were quantified using ELISA kits obtained from R&D Systems (Minneapolis, MN, USA), following the prescribed experimental protocols.

#### 2.3.4 DNA Extraction and 16S rRNA Amplicon Sequencing

To characterize the gut microbial community, metagenomic DNA isolation from fecal samples was performed using the FastDNA SPIN Kit for Soil (MP Biomedicals, Santa Ana, CA, USA) according to the prescribed manufacturer's instructions. The metagenomic library was prepared according to the Illumina 16S Metagenomic Sequencing Library Preparation guide. The hypervariable V3–V4 region of the bacterial 16S rRNA gene was targeted for PCR amplification using standardized primers. Following amplification, the resulting products were quantified and normalized using the Quanti‐iT PicoGreen dsDNA Assay Kit (Invitrogen, Carlsbad, CA, USA). The integrity and size distribution of the prepared libraries were verified using a TapeStation DNA ScreenTape D1000 system (Agilent Technologies, Palo Alto, CA, USA). High‐throughput sequencing was performed on the Illumina MiSeq platform (Illumina, San Diego, CA, USA) utilizing a 2 × 300 bp paired‐end configuration to ensure comprehensive coverage of the V3–V4 region.

#### 2.3.5 Bioinformatics Analysis of Microbiota Data

A rigorous bioinformatics characterization of the fecal microbial community was executed through the QIIME2 (Quantitative Insights Into Microbial Ecology 2) platform (version 2020.8) and the R‐based vegan package (Bolyen et al. 2019; Oksanen et al. 2007). In this 16S rRNA metagenome analysis, we used one of the commonly used method, the Operational Taxonomic Unit (OTU), which considers sequences with over 97% similarity as the same taxonomic unit (Eren et al. 2015). Taxonomic assignment reached species‐level precision through a Bayesian approach utilizing the NCBI RefSeq database (version 2019.2.1) (Gao et al. 2017). To determine within‐sample richness and evenness, Shannon and Pielou indices were calculated. Between‐sample variations (beta diversity) were assessed using both phylogenetic (unweighted UniFrac) and non‐phylogenetic (Bray–Curtis) distances, followed by ordination via PCoA. To identify biomarkers characteristic of each experimental group, we utilized the Linear Discriminant Analysis Effect Size (LEfSe) algorithm to detect statistically significant microbial signatures (Segata et al. 2011). Lastly, the functional landscape of the fecal microbiome was inferred using the PICRUSt2 algorithm in conjunction with the MetaCyc database (Douglas et al. 2020).

### 2.4 Statistical Analyses

All chemical analyses were performed in biological triplicate with three to five technical replicates per sample. For in vitro assays, biological triplicates were performed with each conducted in technical triplicate. In vivo experiments utilized mice randomly assigned to groups (*n* = 6 per group). Quantitative data are reported as means ± SD. Statistical significance was determined through Student's *t*‐tests or one‐way ANOVA, depending on the number of experimental cohorts being compared. For the microbiota analysis, alpha diversity indices were compared using the Mann–Whitney *U* test. The statistical significance of the community‐wide differences between groups was evaluated using Permutational Multivariate Analysis of Variance (PERMANOVA). A *p*‐value of < 0.05 was considered statistically significant, with specific values (*p* < 0.01 and *p* < 0.001) noted where applicable.

## 3 Results

### 3.1 Characteristic Compositions of “Mubong” Peel and Flesh Extracts

The phytochemical characterization of the “Mubong” extract identifies a robust and complex profile of bioactive metabolites, with naringin, neohesperidin, and d‐limonene serving as the primary chemical markers. The physicochemical properties, flavonoid compositions, and antioxidant potentials of Mubong flesh and peel extracts are presented in Tables 2 and 3. Physicochemical analysis of the flesh revealed a TSS content of 13.84°Brix ± 0.78°Brix and a TA of 2.23% ± 0.10%. The sugar composition is defined by glucose (27.29 ± 2.63 mg/mL), fructose (27.07 ± 2.57 mg/mL), and sucrose (27.07 ± 2.65 mg/mL), while citric acid (22.23 ± 2.24 mg/mL) represented the major organic acid constituent.

**TABLE 2:** Physicochemical properties of flesh juices in “Mubong” fruit samples.

Character/compound	Content
TSS (°Brix)	13.84 ± 0.78
Glucose (mg/mL FJ)	27.29 ± 2.63
Fructose (mg/mL FJ)	27.07 ± 2.57
Sucrose (mg/mL FJ)	27.07 ± 2.65
TA (%)	2.23 ± 0.10
Citric acid (mg/mL FJ)	22.23 ± 2.24

Abbreviations: FJ, fresh juice; TA, titratable acidity; TSS, total soluble solids.

**TABLE 3:** Phytochemical composition and antioxidant activity of the flesh and peel of “Mubong” fruit sample.

Character/compound	Content (flesh)	Content (peel)
Rutin (mg/100 g)	43.90 ± 2.60	84.66 ± 19.72
Narirutin (mg/100 g)	64.49 ± 3.24	158.28 ± 1.55
Naringin (mg/100 g)	634.01 ± 34.83	2730.66 ± 93.90
Hesperidin (mg/100 g)	19.14 ± 2.03	110.42 ± 9.25
Neohesperidin (mg/100 g)	122.88 ± 7.99	1493.85 ± 82.67
Nobiletin (mg/100 g)	ND	7.70 ± 0.16
DPPH (IC_50_, mg/mL)	3.86 ± 0.07	1.78 ± 0.02
ABTS (IC_50_, mg/mL)	1.01 ± 0.02	0.02 ± 0.00
FRAP (mg AAE/100 g)	232.13 ± 38.21	1057.07 ± 45.42
TPC (mg GAE/100 g)	248.26 ± 5.16	774.06 ± 6.49
TFC (mg QE/100 g)	21.67 ± 0.32	172.34 ± 1.14

Abbreviations: AAE, ascorbic acid equivalents; ABTS, 2,2′‐azino‐bis(3‐ethylbenzothiazoline‐6‐sulfonic acid); DPPH, 2,2‐diphenyl‐1‐picrylhydrazyl; FRAP, ferric reducing antioxidant power; GAE, gallic acid equivalents; ND, not detected; QE, quercetin equivalents; TFC, total flavonoid contents; TPC, total phenolic contents.

A detailed examination of the flavonoids identifies naringin as the predominant phytochemical, with levels recorded at 634.01 ± 34.83 mg/100 g in the flesh and 2730.66 ± 93.90 mg/100 g in the peel. Neohesperidin follows as a major bioactive component, particularly concentrated within the peel extract at 1493.85 ± 82.67 mg/100 g. Other significant flavonoids contributing to the extract's complexity include narirutin (64.49 mg/100 g in the flesh and 158.28 mg/100 g in the peel), hesperidin (19.14 and 110.42 mg/100 g, flesh and peel, respectively), and rutin (43.90 and 84.66 mg/100 g, flesh and peel, respectively), with nobiletin (7.70 ± 0.16 mg/100 g) uniquely detected in the peel.

The VOC of “Mubong” peel and flesh were characterized via GC–MS, identifying a total of 40 compounds that accounted for 95.45% and 96.02% of the total peak areas, respectively (see Table S1). In both tissues, the monoterpene d‐limonene was identified as the predominant constituent, representing 69.06% of the peel and 88.71% of the flesh. While the flesh was characterized by a relatively simplified profile dominated by d‐limonene and β‐myrcene (2.76%), the peel exhibited significantly greater phytochemical diversity, containing 37 identified compounds. Major secondary components in the peel included linalool (7.82%), β‐myrcene (3.88%), β‐ocimene (3.06%), sabinene (2.23%), and carvone (2.16%). Notably, several bioactive constituents, including α‐tujene, sabinene, carvone, and perilla aldehyde, were detected exclusively in the peel. Furthermore, a variety of minor sesquiterpenes, such as β‐caryophyllene, humulene, and germacrene‐D, were present at concentrations below 0.2% only within the peel tissue, distinguishing its chemical complexity from that of the flesh.

The TPC was measured to be 248.26 and 774.06 mg GAE/100 g, while the TF content was 21.67 and 172.34 mg QE/100 g, in the flesh and peel respectively. These concentrations correspond to significant ferric reducing antioxidant power (FRAP), reaching 1057.07 and 232.13 mg AAE/100 g, and robust radical scavenging activity as demonstrated by IC_50_ values for DPPH (1.78 and 3.8 mg/mL) and ABTS (0.02 and 1.01 mg/mL), in the peel and flesh respectively. Based on the preliminary phytochemical screening, which revealed a significantly higher concentration of bioactive metabolites in the peel compared to the flesh, the “Mubong” peel extract (MPE) was selected for subsequent experiments.

### 3.2 Anti‐Inflammatory Effect of MPE on LPS‐Stimulated RAW264.7 Cells

**FIGURE 2:**
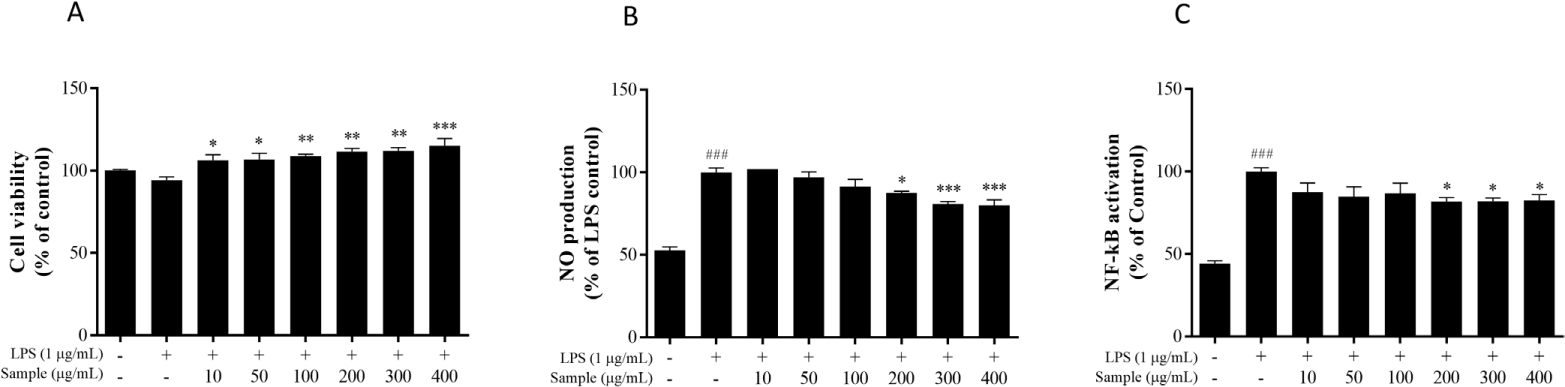
Effect of MPE treatment on cell viability (A), NO production (B) of LPS stimulated RAW264.7 cells, and (C) NF‐κB activation of LPS stimulated RAW‐Blue cells. Data are presented as the mean ± SD of three independent experiments (*n* = 3). Statistical significance was determined by one‐way ANOVA followed by Tukey's post hoc test. ^###^ *p* < 0.001 vs. the control group (no treatment); *p < 0.05, **p < 0.01, and ***p < 0.001 vs. the LPS‐only treated group.

The anti‐inflammatory effect of MPE obtained by ethanol (70%) extraction was investigated on LPS‐stimulated RAW 264.7 cells. To identify a safe and effective concentration for subsequent experiments, we first investigated the potential cytotoxic effects of MPE on RAW 264.7 cells using an MTT assay. Cells were pretreated with varying concentrations of MPE (0–400 μg/mL) in the presence of 1 μg/mL LPS. While LPS treatment resulted in a slight decline in viability that did not reach statistical significance, MPE demonstrated a cytoprotective effect, enhancing the viability of cells subsequently exposed to LPS (Figure 2A). Our results indicated that MPE concentrations up to 400 μg/mL were not cytotoxic, making them suitable for further evaluation of anti‐inflammatory properties. Consistent with these findings, MPE treatment at higher concentrations (200–400 μg/mL) led to a significant, dose‐dependent attenuation of nitric oxide (NO) production in LPS‐stimulated macrophages (*p* < 0.05) (Figure 2B).

NF‐κB activation was measured following treatment with various concentrations of MPE (0–400 μg/mL) and subsequent stimulation with LPS (1 μg/mL). As shown in Figure 2C, LPS treatment alone significantly increased NF‐κB activation compared to the untreated control group (*p* < 0.001). Co‐treatment with the sample resulted in suppression of NF‐κB/AP‐1‐dependent transcriptional activity at higher concentrations of MPE. Notably, significant inhibition was recorded at concentrations of 200–400 μg/mL (*p* < 0.05 vs. LPS‐treated group), indicating that the MPE attenuates LPS‐induced activation of NF‐κB/AP‐1‐dependent transcriptional activity.

### 3.3 Efficacy of MPE on DSS‐Induced Colitis Symptoms in Mice

**FIGURE 3:**
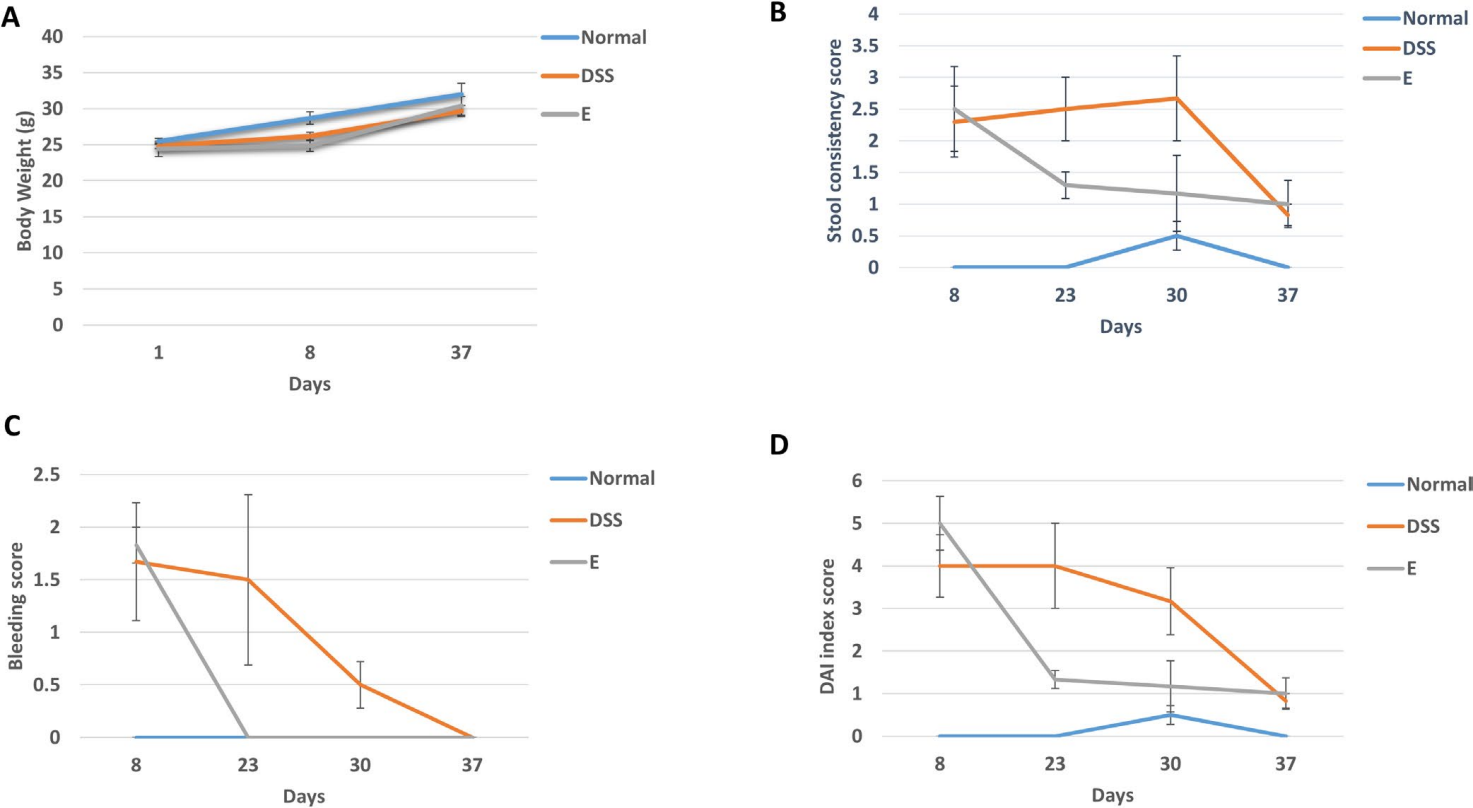
Effects of MPE administration on (A) body weight, (B) stool consistency, (C) bleeding score, (D) DAI index of DSS‐induced colitis mice. Normal = control group; DSS = DSS‐induced group; E = DSS‐induced mice treated with MPE.

Following a one‐week induction period of 2.5% DSS solution in place of drinking water, mice were administered MPE (400 mg/kg) daily for a duration of four weeks. Analysis of DAI components and composite scores (Figure 3A–D) showed that the DSS treatment successfully induced severe colitis, characterized by suppressed body weight gain (final weight 29.67 g vs. normal 32.05 g) and peak DAI score of 4.00 on Day 8, driven by severe diarrhea and rectal bleeding relative to the untreated control group (DAI 0.00). The observed weight gain during the DSS induction period (1.33 g for the DSS group and 0.45 g for the E group) was notably lower than the 3.22 g recorded in the normal control group. The MPE treated (E group) mice demonstrated a potent mitigating effect of the treatment across all metrics despite successful colitis induction (initial DAI 5.00 on Day 8): their body weight trajectory was closer to Normal (30.38 g final weight), their stool consistency rapidly improved, and critically, their rectal bleeding score dropped to zero by Day 23, remaining there for the remainder of the study. This rapid resolution of cardinal symptoms resulted in a dramatic and sustained drop in the E group's overall DAI score, falling to 1.33 by Day 23 and remaining low, clearly demonstrating the MPE treatment's efficacy in mitigating overall disease severity compared to the resolving, but still significantly affected DSS treated group.

**FIGURE 4:**
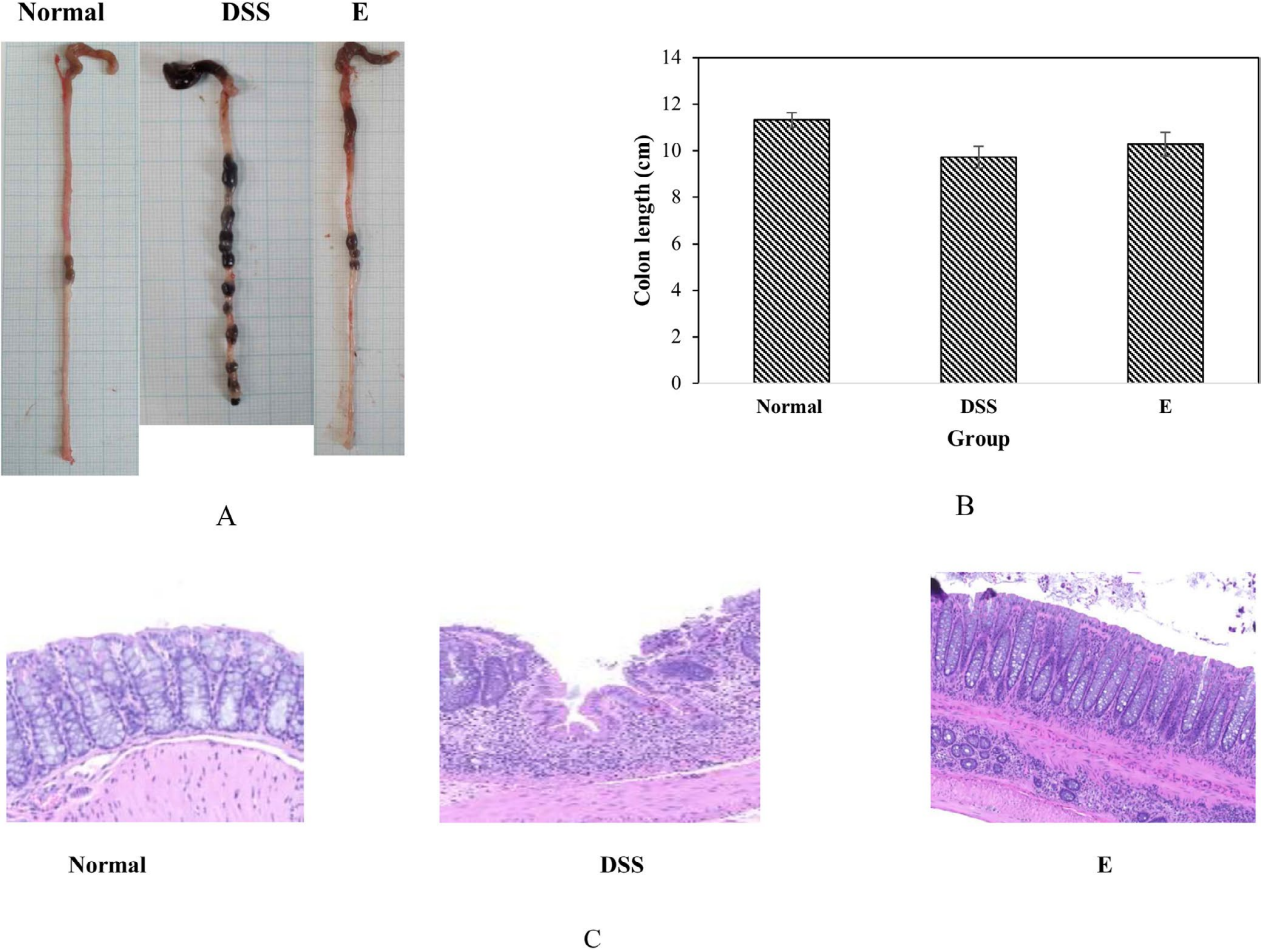
Effect of MPE treatment on gross and histopathological colon morphology. (A) A representative gross picture of colons. The background grid consists of major gridlines representing 1 cm intervals, used for morphological calibration. (B) Measurement of colon length (cm). Data are presented as mean ± SD (*n* = 6). (C) Representative images of H&E‐stained colon sections (magnification = 400×). The control group shows intact mucosal architecture. The DSS‐only group exhibits mucosal epithelial damage, crypt loss, and inflammatory cell infiltration. E group shows maintenance of the mucosal epithelium and crypt structure. Normal = Control group; DSS = DSS‐induced group; E = DSS‐induced mice treated with MPE.

To study the therapeutic effect of MPE at the organ level, mice were immediately dissected following euthanasia, and their colons were excised for observation. The dissected colons and their length from untreated mice (Normal group), DSS‐treated mice (DSS group), and DSS + MPE‐treated mice (E group) are shown in Figure 4A,B. The colon from the normal mice appears healthy, with a pale pink color and a smooth, uninflamed appearance; whereas, the colon from the DSS‐treated mice is visibly inflamed, shortened, and contains black fecal pellets, which are indicative of gastrointestinal bleeding and severe inflammation (Chassaing et al. 2014; Zhou et al. 2023). This morphology is consistent with a successful induction of colitis. The E group mice colon shows an improvement compared to the DSS group. It is longer, less inflamed, and the presence of fecal pellets is much reduced. Its appearance is closer to that of the normal colon, suggesting that the MPE had a protective and therapeutic effect. Figure 4C shows the cross‐sections of the colon tissue, stained using H&E staining to highlight cellular structure and assess the histopathological changes. The normal colon tissue displays intact crypt architecture, with well‐organized glands and a healthy mucosal layer, representing the healthy baseline for the tissue. However, the DSS‐treated tissue shows extensive damage. The crypts are severely disrupted and shortened, and there is a significant infiltration of inflammatory cells, indicating a robust inflammatory response and mucosal ulceration. This histological damage confirms the presence of severe colitis. The tissue from the E group shows a marked improvement. The crypt structure is largely preserved, with reduced inflammatory cell infiltration and a healthier mucosal surface. While not perfectly normal, the tissue shows a clear recovery from the damage observed in the DSS group.

### 3.4 Impact of MPE on Short‐Chain Fatty Acids (SCFAs) and Inflammatory Cytokines

**FIGURE 5:**
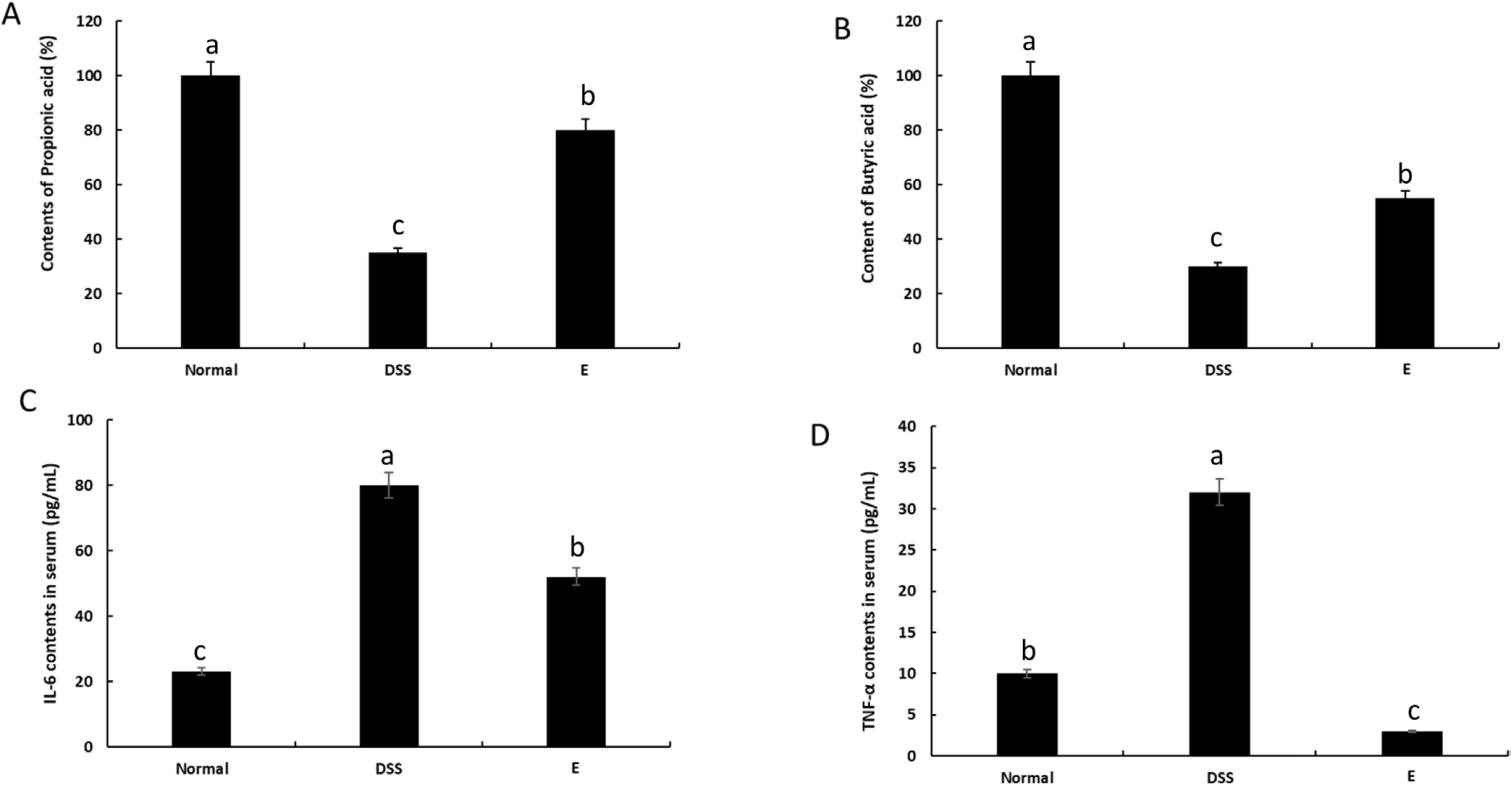
Quantitative analysis of fecal short‐chain fatty acids (SCFAs) and serum cytokines. The figure evaluates the metabolic and inflammatory status across groups (A) Propionic acid and (B) Butyric acid contents in feces, expressed as a percentage (%) relative to the normal group; (C) Interleukin‐6 (IL‐6) and (D) Tumor Necrosis Factor‐alpha (TNF‐α) concentrations in serum, expressed in picograms per milliliter (pg/mL). All data are presented as mean ± standard deviation (*n* = 6 per group). Statistical significance was determined using one‐way ANOVA followed by Tukey's post hoc test for multiple comparisons. Different lowercase letters (a, b, c) above the bars indicate significant differences between groups at *p* < 0.05.

The DSS‐treated group showed a severe drop in both propionic acid (to approximately 35% of normal levels) and butyric acid (to about 30% of normal levels) (Figure 5A,B). However, the administration of MPE effectively mitigated this effect, helping to restore these critical metabolites. The MPE treated group's propionic acid levels rose significantly to approximately 80% of normal, while butyric acid levels increased to over 50% of the normal baseline.

In addition to restoring SCFAs, MPE treatment was associated with the modulation of key pro‐inflammatory signaling molecules. Induction of colitis via DSS challenge led to a profound elevation in serum pro‐inflammatory cytokines. Specifically, IL‐6 concentrations in the DSS‐treated group surged to approximately ~80 pg/mL—representing a fourfold increase over the healthy baseline of ~22 pg/mL (Figure 5C). A similar trend was observed for TNF‐α, which escalated to over ~30 pg/mL, a threefold rise compared to the ~10 pg/mL recorded in the normal control group (Figure 5D). The inflammatory surge was markedly suppressed following MPE intervention. IL‐6 levels were reduced to ~52 pg/mL, whereas TNF‐α concentrations plummeted to near‐baseline levels (~3 pg/mL). This dramatic downregulation of TNF‐α, in particular, underlines the anti‐inflammatory efficacy of MPE in neutralizing the mediators that drive colonic injury.

### 3.5 Impact of MPE on Gut Microbiota of DSS‐Induced Colitis Mice

Given that dietary intake can directly modulate the taxonomic distribution of gut microbiota, we analyzed the shifts in microbial populations and the regulatory effects of MPE on intestinal microbiota. To investigate the impact of MPE on the gut microbiome of mice with DSS‐induced colitis, the V3–V4 hypervariable regions of the 16S rRNA gene were sequenced. A total of 8,752,703 sequences were recovered from 75 samples, providing a robust dataset for the subsequent characterization of taxonomic shifts and community structure.

#### 3.5.1 Diversity

**FIGURE 6:**
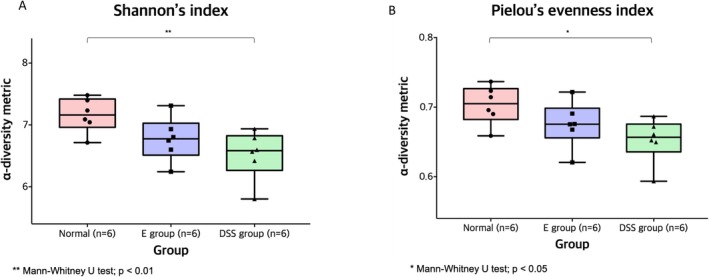
Alpha diversity indices of fecal samples in each group: Shannon's index (A) and Pielou's evenness index (B). Alpha diversity was assessed to evaluate the internal complexity of the microbial community in each group (*n* = 6). Shannon's index, representing both species richness and evenness, and Pielou's evenness index, representing the distribution of individual species. The DSS‐induced colitis group exhibited a significant decrease in microbial diversity and evenness compared to the Normal group (**p < 0.01 and *p < 0.05, respectively). Statistical significance was determined using the non‐parametric Mann–Whitney *U* test.

Alpha (α) diversity is an index that represents the diversity of microorganisms within a sample, largely consisting of two key components: richness and evenness. Richness refers to the number of different microbial species present in the sample, while evenness describes how uniformly these microorganisms are distributed. For this study, the analysis was performed using Shannon's index and Pielou's evenness index (Pielou 1966; Shannon 1948). The Shannon's index for the gut microbiota in the Normal group was higher than in the DSS group. Following the induction of colitis with DSS, a reduction in microbial diversity was observed. Administration of MPE was associated with a gradual increase in diversity indices (Figure 6A). Similarly, the Pielou's evenness index showed that α‐diversity in the DSS group was decreased compared to the Normal group. It is presumed that the microbiota's evenness, which was diminished by the induction of colitis, gradually recovered over MPE administration (Figure 6B). However, both the Shannon's and Pielou's evenness indices showed only a weak, insignificant correlation with both the weight of the thymus and the length of the colon. The figures provided illustrate these relationships, with the blue line representing the regression line and the red lines indicating the 95% confidence band (see Figure S1). While MPE treatment modulated the microbial landscape, the lack of a significant correlation between Shannon's or Pielou's indices and physical markers (colon length and thymus weight) suggests that the overall alpha diversity may not be a direct proxy for disease severity in this model. Rather, the therapeutic benefits of MPE appear to be driven by compositional shifts—specifically the enrichment of key obligatory anaerobes and the suppression of facultative anaerobes—rather than broad changes in species richness or evenness.

**FIGURE 7:**
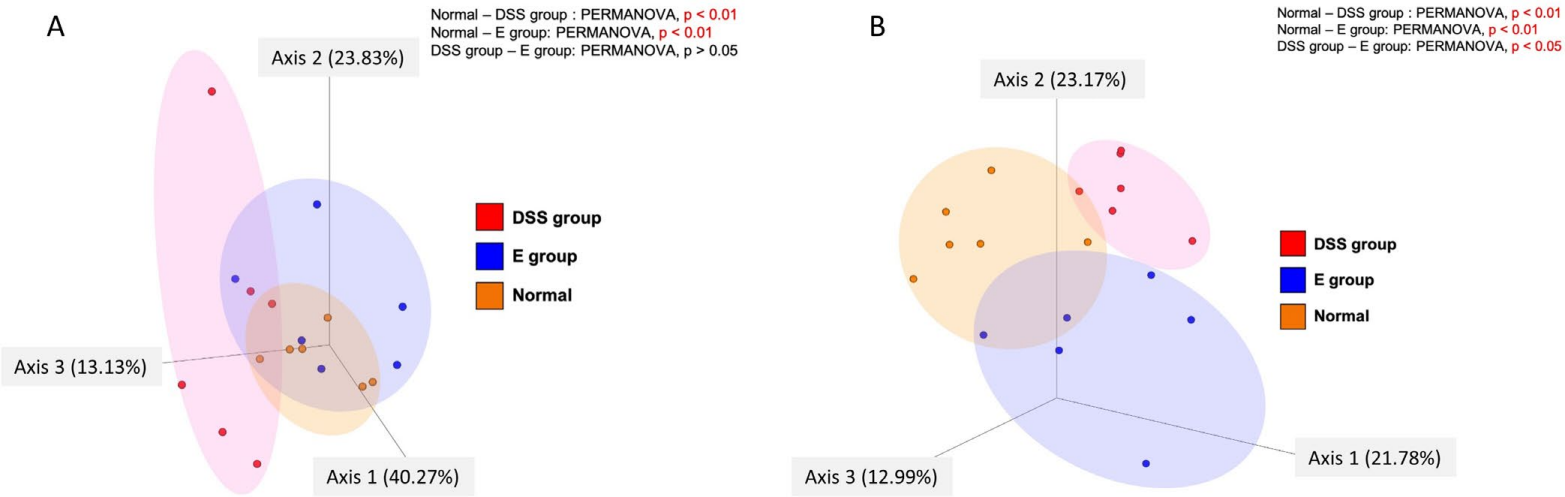
Beta diversity of fecal microbial communities. Principal coordinate analysis (PCoA) based on Unweighted UniFrac distance (A) and Bray–Curtis dissimilarity (B) (*n* = 6). The percentage of total variance explained by each axis is indicated in parentheses on the axes. Statistical differences in microbial community composition were evaluated using PERMANOVA. Significant separation was observed between the Normal and DSS groups (*p* < 0.01), while the MPE‐treated group showed a significant shift in composition compared to the DSS‐only group in the Unweighted UniFrac analysis (*p* < 0.05).

Beta (β) diversity is an index that represents the diversity of microorganisms between samples, and it's broadly classified into two types of approaches. Unweighted approaches such as Unweighted UniFrac (Lozupone and Knight 2005), calculated based on the simple presence or absence of microorganisms in a sample, and weighted approaches such as Bray–Curtis dissimilarity (Bray and Curtis 1957), calculated by weighting the abundance of microorganisms in the sample. Principal Coordinate Analysis (PCoA) was performed using two metrics to assess beta diversity. Analysis using Unweighted UniFrac distance showed that while both the DSS and E groups had distinct microbial compositions compared to the Normal group, the E group appeared slightly closer in composition to the Normal group (Figure 7A). When the analysis was weighted by microbial abundance using Bray–Curtis Dissimilarity, the PCoA similarly revealed clustered microbial communities (Figure 7B). For both metrics, a subsequent multivariate ADONIS test confirmed that the group variable (Normal, E, and DSS) had the most significant statistical influence, indicating that the observed differences and clustering in both PCoA plots are primarily driven by the effect of the group.

#### 3.5.2 Comparative Analysis

**FIGURE 8:**
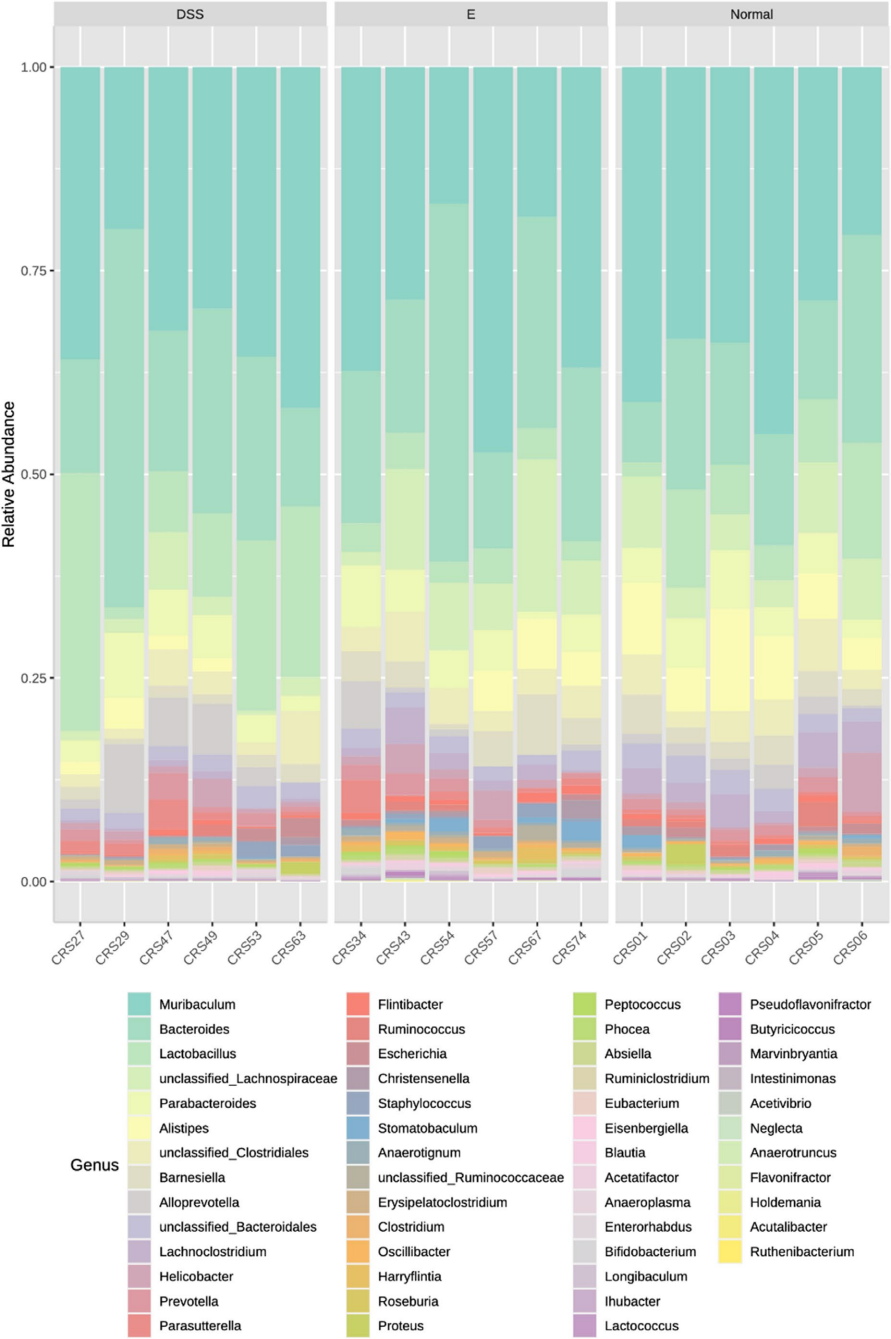
Distribution of gut microbial genera across experimental groups. The bar plot represents the relative abundance of bacterial genera for individual samples within the Normal, DSS, and E (MPE‐treated) groups (*n* = 6). Each color represents a specific genus, as indicated in the legend. The plot illustrates the shift in community composition from a diverse population of obligatory anaerobes in the Normal group to a pronounced dysbiosis in the DSS group, followed by partial restoration of the taxonomic profile in the E group.

**FIGURE 9:**
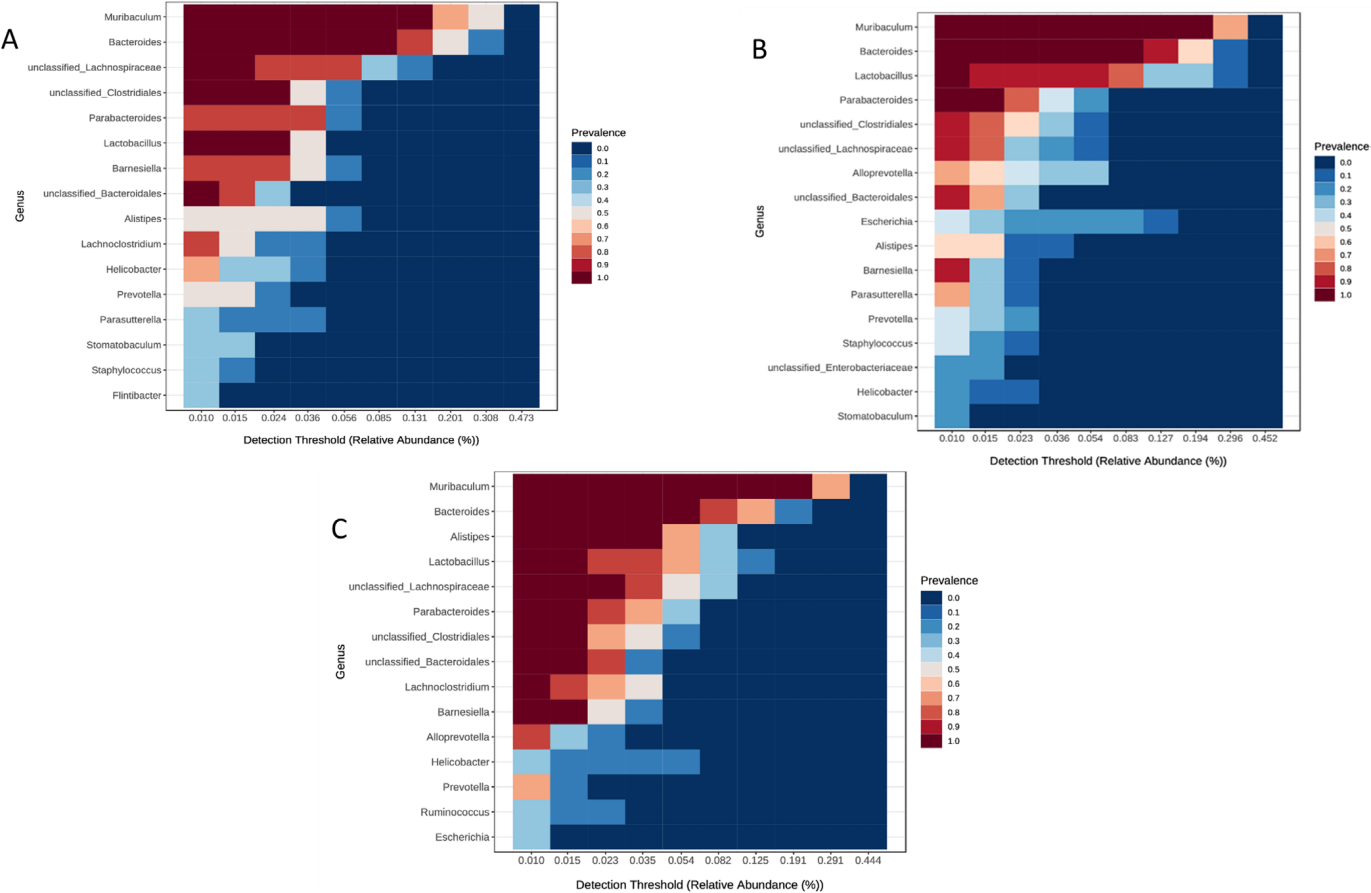
Heatmap of the core microbiota associated with E group (MPE‐treated) (A), DSS group (B), and normal group (C). The heatmap illustrates the prevalence of bacterial genera across varying relative abundance detection thresholds (*x*‐axis). The color gradient from blue (0.0) to red (1.0) indicates the proportion of samples within each group that harbor a specific genus at the corresponding abundance level. Taxonomic identification is provided at the genus level on the *y*‐axis, with major taxa including *Muribaculum*, *Bacteroides*, and *Lachnoclostridium*.

Gut microbiota compositional profile were further analyzed at the genus levels. The genera identified in each group are represented in a bar plot at Figure 8. The core microbiota, defined as genera exhibiting high prevalence within each group, was analyzed across each group using prevalence scores derived from a heatmap, where prevalence indicates the fraction of samples exceeding a defined abundance detection threshold (Figure 9). For example, in both E group and DSS group, applying a threshold of 0.036 (3.6%) or higher, *Bacteroides* showed a prevalence of 1.0, indicating its presence at 3.6% or more in all samples; however, *Lactobacillus* showed a prevalence of 0.5 in E group (3.6% or more in 50% of samples), while *Parabacteroides* showed a prevalence of 0.4 in the DSS group (3.6% or more in 40% of samples). On the other hand, in the Normal group analysis utilizing a higher threshold of 0.054 (5.4%) or higher, *Bacteroides* also achieved a prevalence of 1.0, and *Alistipes* exhibited a prevalence of 0.6 (5.4% or more in 60% of samples). The core microbiota identified in each group show clear differences: in the MPE treated group, there is a tendency for *Lachnoclostridium*, unclassified *Lachnospiraceae*, and unclassified *Clostridiales* to increase compared to the DSS group, while *Parabacteroides* tends to decrease. Furthermore, compared to the control group, both the MPE treated and DSS‐induced groups show a marked decrease in the abundance of both *Alistipes* and *Barnesiella*.

Relative abundance analysis is the process of calculating statistical or pattern‐based differences in microbial distribution between groups. The analysis primarily uses various methods, with a focus on those that produce statistically significant results. The Linear Discriminant Analysis Effect Size (LEfSe) analysis, applying a statistical cutoff of FDR ≤ 0.05, didn't identify any genera showing statistically significant group‐specific enrichment. However, the box plots (see Figure S2) display major bacterial genera that exhibited distinctive distribution patterns across the groups, with a higher Linear Discriminant Analysis (LDA) score suggesting the corresponding genus is more evenly and abundantly distributed within a specific group. *Alistipes*, a beneficial bacterium known to decrease in those with intestinal diseases like ulcerative colitis (Lepage et al. 2011), was markedly decreased in both the E and DSS groups. *Lactobacillus*, widely recognized as a beneficial gut bacterium and reported to negatively correlate with colon inflammation (Ju et al. 2024; Lee et al. 2022), paradoxically increased in the DSS group in this study. *Lachnoclostridium* showed a significant reduction in the DSS group but trended toward recovery upon MPE treatment, despite conflicting results in prior research. *Acetatifactor*, which produces beneficial short‐chain fatty acids (SCFAs) (Pfeiffer et al. 2012), was significantly reduced in the DSS group and showed a gradual recovery with MPE treatment. *Erysipelatoclostridium*, which tends to increase in patients with intestinal diseases such as Crohn's disease (Mancabelli et al. 2017), showed a marked increase in both the DSS and E groups. *Parasutterella* was significantly elevated with DSS induction and showed a gradual decrease following MPE treatment. *Lactococcus* was more prevalent in the Normal group. *Barnesiella*, a beneficial bacterium known to decrease in individuals with intestinal diseases (Kulagina et al. 2012; Ubeda et al. 2013), was significantly reduced due to DSS induction but increased in MPE treated mice.

**FIGURE 10:**
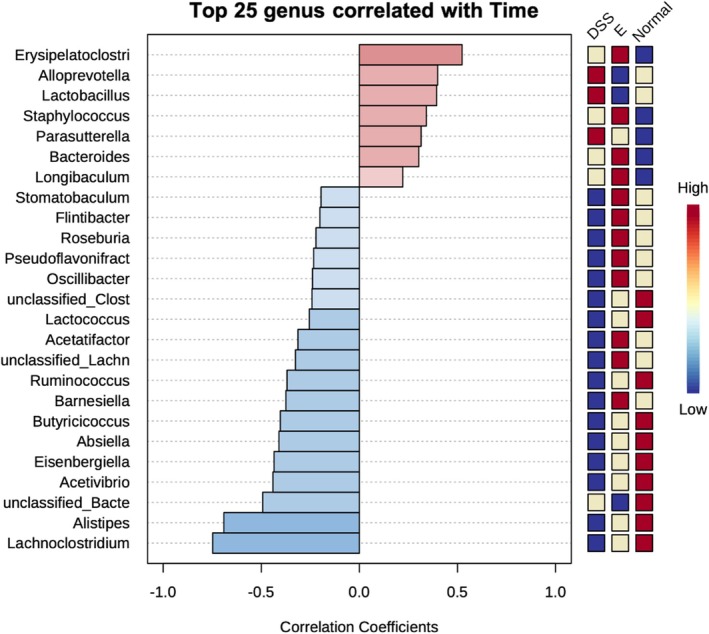
Taxonomic correlation analysis of gut microbial genera. Bar plot illustrating the top 25 bacterial genera showing the strongest correlation with the experimental progression (Normal → DSS → E). The *x*‐axis represents the Spearman's rank correlation coefficients; red bars indicate a positive correlation (increasing abundance toward the E‐treatment group), while blue bars indicate a negative correlation (decreasing abundance toward the E‐treatment group). The accompanying heatmap indicates the relative abundance of each genus within the DSS, E, and Normal groups. Although these genera represent the most prominent longitudinal trends identified via Spearman's analysis, no taxa reached statistical significance after Benjamini–Hochberg false discovery rate (FDR) correction (*q* > 0.05).

Assuming a longitudinal study design in which colitis is induced in the normal group by administering DSS, followed by treatment with MPE, we investigated the pattern of gut microbial abundance across the Normal → DSS → E groups using correlation analysis. The correlation coefficients in the plot (Figure 10) indicate how abundance changed with MPE treatment: a positive correlation suggests abundance increased toward the E group, while a negative correlation suggests it decreased. Specifically, *Erysipelatoclostridium*, *Alloprevotella*, and *Lactobacillus* showed an increasing pattern toward the E group, while *Lachnoclostridium* and *Alistipes* showed a decreasing pattern toward the E group. These findings suggest that in this intestinal inflammation colitis model, administration of the MPE may lead to a marked decrease in genera like *Lachnoclostridium* and *Alistipes*, while genera such as *Erysipelatoclostridium* and *Alloprevotella* may slightly increase. However, it is important to note that, while individual taxa did not meet the strict FDR‐adjusted significance threshold, the Spearman's rank correlation analysis revealed clear directional shifts in the microbial community, particularly for genera associated with intestinal homeostasis such as *Lactobacillus* and *Alistipes*.

#### 3.5.3 Function Analysis

Function analysis is a method that infers gene families within the microbiome based on genes generally possessed by microbes, which allows for the ultimate prediction of active metabolic pathways. Although this approach is limited because its reliance on reference genomes prevents the resolution of strain‐specific functionality and may introduce bias from genotype–phenotype discrepancies, it remains academically recognized for its predictive power (Douglas et al. 2020). Metabolic pathway prediction based on PCoA showed no significant differences in the overall distribution of metabolic pathways among the E, DSS, and Normal groups, with no pathways showing group‐specific enrichment at a statistical cutoff of FDR ≤ 0.05 (see Figure S3). Despite the lack of statistical significance (indicated by a higher LDA score meaning a more even and abundant distribution within a group), several key pathways displayed distinct patterns: protein synthesis pathways, typically active in health, were highly expressed in the Normal group, decreased in the DSS group, and appeared to recover with MPE treatment. Similarly, the menaquinol biosynthesis pathway (related to anticoagulation) was markedly reduced in the DSS group but predicted to recover following MPE treatment. Conversely, the heme biosynthesis pathway was highest in the DSS group, possibly due to intestinal bleeding from colitis induction, and the DSS group also showed overall higher expression of carbohydrate degradation pathways, including sucrose, fucose, and lactose metabolism, though the cause of this increase requires further investigation.

## 4 Discussion

Citrus fruit peels have a long‐standing traditional use as an anti‐inflammatory agent (Yu et al. 2018). This therapeutic effect is partly attributed to their richness in beneficial metabolites, such as phenolic compounds, volatile organic compounds, and flavonoids including polymethoxyflavones (PMFs), such as nobiletin and tangeretin (Ho and Kuo 2014; Malleshappa et al. 2018; Musumeci et al. 2020; Wang et al. 2018). Limonene was identified as the predominant volatile organic compound (VOC) in “Mubong,” accounting for 70% and 88% of the chemical profile in the peel and flesh, respectively. Recent evidence suggests that limonene alleviates ulcerative colitis through synergistic anti‐inflammatory and anti‐oxidative mechanisms, alongside the regulation of the intestinal microenvironment (Xu et al. 2025). The phytochemical profiling (Table 3) revealed that “Mubong” peel extract contained the flavonoids rutin, narirutin, naringin, hesperidin, neohesperidin, and nobiletin. Studies have demonstrated that these compounds are involved in suppressing NF‐κB signaling (Cao et al. 2025; Jie et al. 2025; Li et al. 2021, 2018; Subramanian et al. 2015; Wang et al. 2024), mitogen‐activated protein kinase (MAPK) signaling (Cao et al. 2025; Jie et al. 2025), and improving intestinal barrier function (Cao et al. 2021; Liu, Ding, et al. 2024; Wang et al. 2024; Xiong et al. 2015). PMFs, which are highly concentrated in citrus peels, are established modulators of inflammatory signaling pathways (Fontana et al. 2023; Ho and Kuo 2014). This phytochemical profile is further enriched by other major flavonoids identified in the “Mubong” peel—specifically narirutin, hesperidin, neohesperidin, naringin, and rutin—all of which have been reported to show efficacy in IBD management via gut microbiota modulation (Cao et al. 2021; Ju et al. 2024; Li et al. 2023; Stevens et al. 2022; Xu et al. 2024). Furthermore, previous reports have also indicated that citrus‐derived polyphenols have attenuated mucosal inflammation in DSS‐induced acute colitis models (He et al. 2023). Therefore, the presence of these specific bioactive constituents may contribute to the attenuated inflammatory response and the restoration of microbial diversity observed in our study. Citrus‐derived phytochemicals are well‐documented for their robust antioxidant capacity (Saini et al. 2022; Zou et al. 2016); accordingly, the antioxidant activity exhibited by “Mubong” extracts in this study may further validate the therapeutic potential of citrus bioactives. By intercepting the oxidative‐inflammatory axis, these “Mubong” fruit extracts may provide a critical mechanism for mitigating mucosal damage and restoring intestinal homeostasis. Complementing the therapeutic profile of individual citrus‐derived compounds, citrus peel derivatives—both in extract and powder forms—have been reported to attenuate epithelial barrier defects and dampen inflammatory responses in DSS‐induced colitis (Kim et al. 2025; Tinh et al. 2021).

Building upon this established knowledge, we hypothesize that an extract derived from “Mubong” mandarin peels could similarly exhibit anti‐inflammatory properties and offer a promising therapeutic approach for conditions involving inflammation, specifically focusing on colitis. This study explored the effect of MPE against DSS‐induced UC. The findings suggested that the extract may contribute to reducing symptoms of colitis, regulate the levels of inflammatory factors and short‐chain fatty acids, potentially enhancing the function of intestinal barriers, and regulating the composition of intestinal microbiota.

In DSS‐induced colitis model, body weight change is one of the critical and easily measured parameters used to assess the severity of colitis and the efficacy of therapeutic treatments (Britto et al. 2019). In this study, the observed stabilization of body weight and the reduction in the DAI, which summarizes clinical symptoms such as diarrhea and rectal (Baumgart and Sandborn 2007) suggests that MPE may attenuate the acute symptoms of ulcerative colitis. These clinical improvements, alongside the preservation of colon length, are consistent with a reduction in mucosal damage and may reflect the extract's potential to support intestinal function (Chassaing et al. 2014). The observation that MPE administration was associated with a reduction in DAI scores and the maintenance of body weight suggests a protective role for the extract against the acute phase of DSS‐induced injury. Specifically, the rapid resolution of rectal bleeding and the stabilization of stool consistency in the E group may reflect the extract's ability to support mucosal healing and mitigate the loss of intestinal barrier function. These clinical improvements are consistent with the anti‐inflammatory effects observed in our in vitro assays and suggest that MPE treatment may mitigate the severity of the inflammatory cascade typically triggered by DSS. While the DSS‐only group showed a degree of natural resolution, the more pronounced recovery of clinical markers in MPE‐treated mice indicates that the phytochemicals present in “Mubong”—such as naringin and neohesperidin—may contribute to accelerating the restoration of gut function.

While standard DSS‐induced colitis protocols using mice aged 7–9 weeks typically reported significant absolute weight loss (Kim et al. 2025), the 6‐week‐old mice (5 weeks at arrival plus 1 week of acclimatization) in this study showed a net weight gain. This is likely attributable to the rapid developmental growth phase of younger animals. However, the DSS and E group exhibited a marked reduction in weight gain (1.33 and 0.45 g, respectively) compared to the normal control group (3.22 g), suggesting that while the young mice maintained a positive growth trajectory, the induction of colitis significantly impaired their normal physiological development. Younger mice are often characterized by a developing immune system, which can result in distinct susceptibility profiles compared to mature adults. However, the fourfold increase in serum IL‐6 and the marked weight gain attenuation demonstrate that 6‐week‐old mice remain highly sensitive to DSS‐mediated injury. The use of this age group allows for the assessment of MPE as a functional food intervention during an active growth phase, a period where gastrointestinal homeostasis is particularly critical.

Pro‐inflammatory cytokines are central to the pathogenesis of IBD, driving the chronic mucosal inflammation that characterizes the condition (Strober and Fuss 2011). Conversely, SCFAs—notably acetate, propionate, and butyrate—serve as essential mediators linking dietary intake and the microbiome to immune homeostasis (Lee et al. 2022; Shin et al. 2023; Venegas et al. 2019). Colitis induced by DSS severely disrupted the gut microbiota, leading to a reduction in the levels of key SCFAs, which are vital for maintaining intestinal homeostasis (Lee et al. 2022). Butyric acid, in particular, is the primary energy source for colon cells, and its depletion is a hallmark of inflammatory bowel disease (Hamer et al. 2008). Clinical observations consistently show that IBD patients exhibit a marked depletion of SCFA levels and their associated producing bacteria (Chulenbayeva et al. 2025). Although statistically significant differences at the level of individual microbial taxa were limited, the overall biological response to MPE treatment was highly consistent across multiple parameters. Suppression of colonic inflammation, as reflected by reduced TNF‐α and IL‐6 levels, was accompanied by restoration of SCFAs, particularly butyrate and propionate, and a gradual recovery of obligate anaerobic bacterial groups. This coordinated response could suggests that MPE might not act on specific microbial taxa in isolation, but rather may be associated with promoting the recovery of the intestinal microenvironment.

The gut microbiota is essential for the maintenance of host homeostasis. Consequently, dysbiosis within the gut microbiome represents a critical factor in the pathogenesis of IBD (Rigottier‐gois 2013). Research indicates that both DSS‐induced colitis models and human patients are characterized by a distinct microbial shift, specifically an increased abundance of pathogenic bacteria alongside a reduction in the relative abundance of beneficial species (Dalal and Chang 2014; Fischler et al. 2025). The therapeutic efficacy of MPE in colitis could be rooted in its ability to restore the anoxic niche of the gut. A healthy colonic environment is characterized by physiological hypoxia, a state maintained by the high oxygen consumption of healthy colonocytes (Rigottier‐gois 2013; Winter et al. 2013). This oxygen‐free environment is essential for the survival of obligatory anaerobes, such as *Faecalibacterium prausnitzii* and *Bacteroides* spp., which are the primary producers of SCFAs like butyrate and propionate (Venegas et al. 2019). However, during DSS‐induced colitis, the surge in pro‐inflammatory cytokines—specifically TNF‐α and IL‐6—disrupts this balance by triggering mucosal damage and vascular permeability (Strober and Fuss 2011). This leads to pathological hyperoxia, where oxygen leaks into the intestinal lumen. While this influx of oxygen is lethal to SCFA‐producing obligatory anaerobes, it provides a competitive advantage to facultative anaerobes that can utilize oxygen for aerobic respiration (Rigottier‐gois 2013). Our findings are consistent with the oxygen hypothesis of intestinal inflammation. Our taxonomic analysis suggests a notable shift in the microbial community composition In the DSS‐induced group, we observed a decline in oxygen‐sensitive obligatory anaerobes, such as *Lachnospiraceae*, *Lachnoclostridium*, and *Acetatifactor*. Conversely, there was a significant increase in the relative abundance of *Lactobacillus* within the DSS‐only group. While initially paradoxical given its probiotic status, this expansion of *Lactobacillus* aligns with the oxygen hypothesis, as many *Lactobacillus* species are facultative anaerobes that can tolerate the microaerobic conditions of an inflamed gut. MPE administration appears to modulate this cycle by suppressing TNF‐α and IL‐6. This may mitigate mucosal inflammation and help preserve the intestinal barrier's integrity, potentially limiting oxygen leakage. The potential restoration of the anoxic niche was suggested by the gradual recovery of *Acetatifactor* and *Lachnoclostridium*, which coincided with increased levels of butyric and propionic acids in MPE‐treated groups. Thus, MPE likely works by shifting the ecological balance away from oxygen‐tolerant groups and back toward the strict anaerobes necessary for a healthy, SCFA‐producing microbiome.

Notwithstanding the significant outcomes reported here, a few methodological constraints warrant acknowledgment to ensure a comprehensive interpretation of the data. First, while we characterized the major phytochemicals, the specific bioactivity of individual compounds—such as naringin, neohesperidin, or d‐limonene—was not validated in isolation; thus, the effects observed may be the result of complex synergistic interactions. Second, the observed shifts in the gut microbiota and the restoration of the anoxic niche remain associative. Future research involving fecal microbiota transplantation (FMT) is necessary to confirm whether these microbial changes are the primary drivers of the anti‐inflammatory effect. Third, the exclusive use of female mice to mitigate group‐housing aggression and baseline stress is another limitation of this study. Given that biological sex can modulate inflammatory responses and gut microbiota composition, future studies incorporating male cohorts are necessary to fully validate the translational applicability of these findings. Finally, while the DSS‐induced colitis model is a well‐established surrogate for human colitis, the findings have limited direct translational applicability to clinical settings. Further clinical trials are necessary to confirm the safety and efficacy of MPE in human populations.

## 5 Conclusions

The present study provides the first comprehensive characterization of the phytochemical profile and therapeutic efficacy of the new citrus hybrid, “Mubong.” Our findings demonstrate that MPE is rich in bioactive secondary metabolites, particularly naringin, neohesperidin, and d‐limonene. In vitro assays showed that MPE exhibited anti‐inflammatory effects by dose‐dependently inhibiting nitric oxide production and modulation of NF‐κB/AP‐1‐dependent transcriptional activity. In the DSS‐induced colitis model, oral administration of MPE was associated with the alleviation of clinical symptoms, as evidenced by the restoration of colon length and a reduction in disease activity index scores. These systemic improvements were accompanied by a reduction in pro‐inflammatory cytokines, TNF‐α and IL‐6. The metagenomic analysis suggests that MPE is associated with changes in the gut microbiota, including an increased abundance of obligatory anaerobes such as members of the *Lachnospiraceae* family and the genera *Lachnoclostridium* and *Acetatifactor*, which were depleted by DSS. This shift coincided with improved levels of beneficial SCFAs, notably butyric and propionic acids. Ultimately, the multi‐target efficacy of MPE identified in this work underscores its viability as a promising candidate for the development of functional food interventions aimed at alleviating chronic gastrointestinal disorders.

## Author Contributions

**Awraris Derbie Assefa:** methodology, writing – original draft, validation, formal analysis. **YoSup Park:** writing – review and editing, supervision. **Seung‐Gab Han:** writing – review and editing, supervision. **Sang Suk Kim:** conceptualization, methodology, writing – original draft, validation, formal analysis. **Jee‐Soo Park:** conceptualization, writing – review and editing, supervision, project administration, funding acquisition.

## Funding

This research was carried out with the support of the “Cooperative Research Program for Agriculture Science and Technology Development” (Project No. PJ01447304) of the National Institute of Horticultural and Herbal Science, Rural Development Administration, Republic of Korea.

## Disclosure

All authors have read and approved the final version of the manuscript. Jee‐Soo Park had full access to all of the data in this study and takes complete responsibility for the integrity of the data and the accuracy of the data analysis.

## Ethics Statement

Animal procedures in this study were performed in accordance with the guidelines approved by the Institutional Review Board of Jeju National University (Protocol Number: 2020‐0030).

## Supporting information


**Figure S1:** Correlation of Shannon's and Pielou's evenness indices with the weight of the thymus and the length of the colon.
**Figure S2:** Major bacterial genera that exhibited distinctive distribution patterns across the groups.
**Figure S3:** Metabolic pathway prediction based on PCoA: Bray–Curtis dissimilarity (A) and Jaccard similarity index (B). Each point represents an individual sample, and samples with similar metabolic pathways are positioned close to one another.

**Table S1:** The composition of volatile organic compounds of Mubong peel and flesh extracts; a = alcohols; b = aldehydes; c = esters; d = hydrocarbons; e = ketones; f = etc.

## Data Availability

The authors confirm that the data supporting the findings of this study are available on request from the corresponding author.
