# Supplementary material for: Ameliorative Effects of Newly Developed Citrus Hybrid “Mubong” Peel Extract on Experimental Colitis and Gut Microbiota Dysbiosis
Source: Food Sci Nutr. 2026 Jul 9;14(7):e72098. doi: 10.1002/fsn3.72098 (PMC13349115; doi:10.1002/fsn3.72098)
Supplement: Supplementary file 1 — Figure S1: Correlation of Shannon's and Pielou's evenness indices with the weight of the thymus and the length of the colon. Figure S2: Major bacterial genera that exhibited distinctive distribution patterns across the groups. Figure S3: Metabolic pathway prediction based on PCoA: Bray–Curtis dissimilarity (A) and Jaccard similarity index (B). Each point represents an individual sample, and samples with similar metabolic pathways are positioned close to one another. [file FSN3-14-e72098-s001.pptx]

## Slide 1
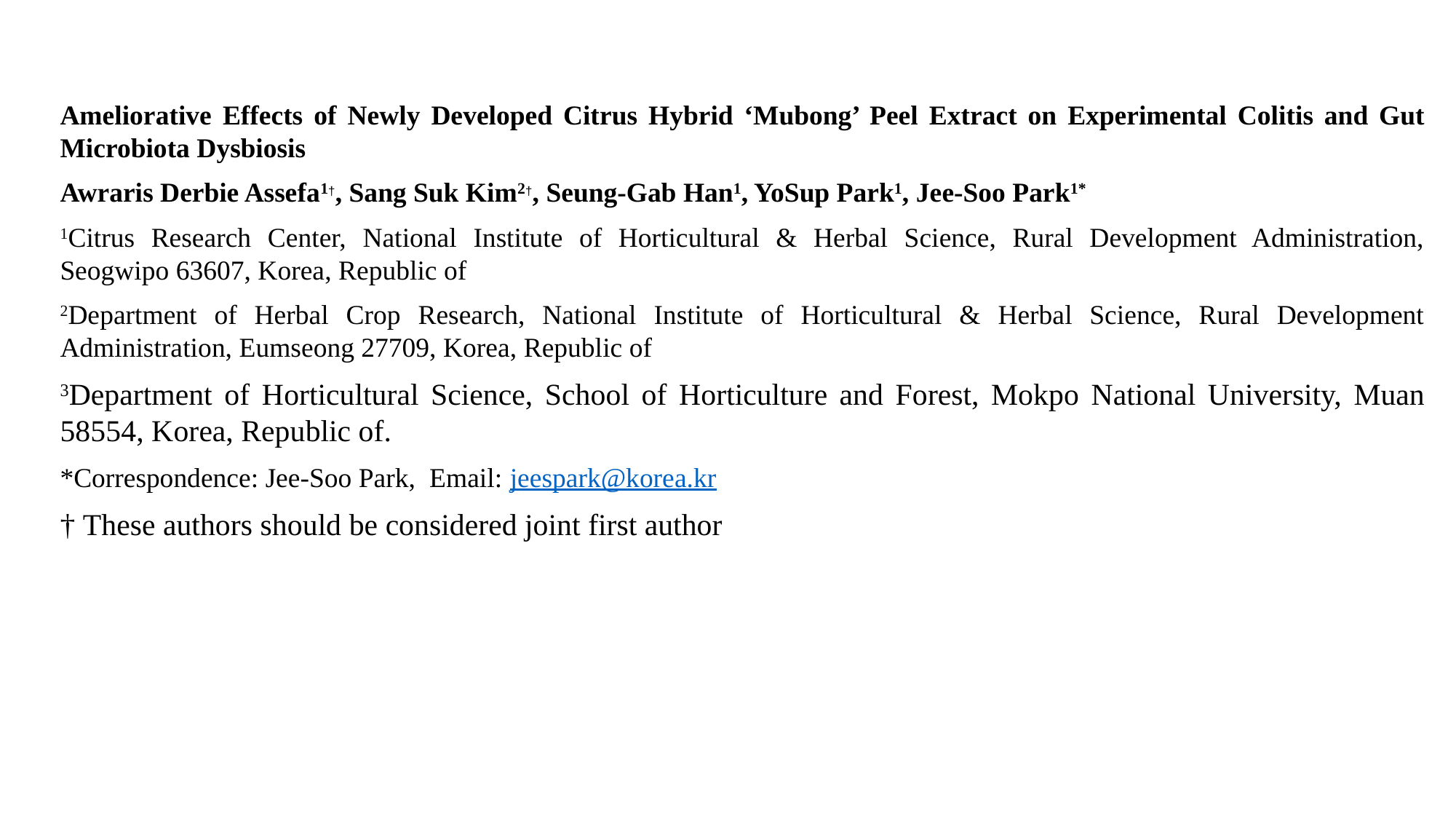

Ameliorative Effects of Newly Developed Citrus Hybrid ‘Mubong’ Peel Extract on Experimental Colitis and Gut Microbiota Dysbiosis
Awraris Derbie Assefa1†, Sang Suk Kim2†, Seung-Gab Han1, YoSup Park1, Jee-Soo Park1*
1Citrus Research Center, National Institute of Horticultural & Herbal Science, Rural Development Administration, Seogwipo 63607, Korea, Republic of
2Department of Herbal Crop Research, National Institute of Horticultural & Herbal Science, Rural Development Administration, Eumseong 27709, Korea, Republic of
3Department of Horticultural Science, School of Horticulture and Forest, Mokpo National University, Muan 58554, Korea, Republic of.
*Correspondence: Jee-Soo Park, Email: jeespark@korea.kr
† These authors should be considered joint first author

## Slide 2
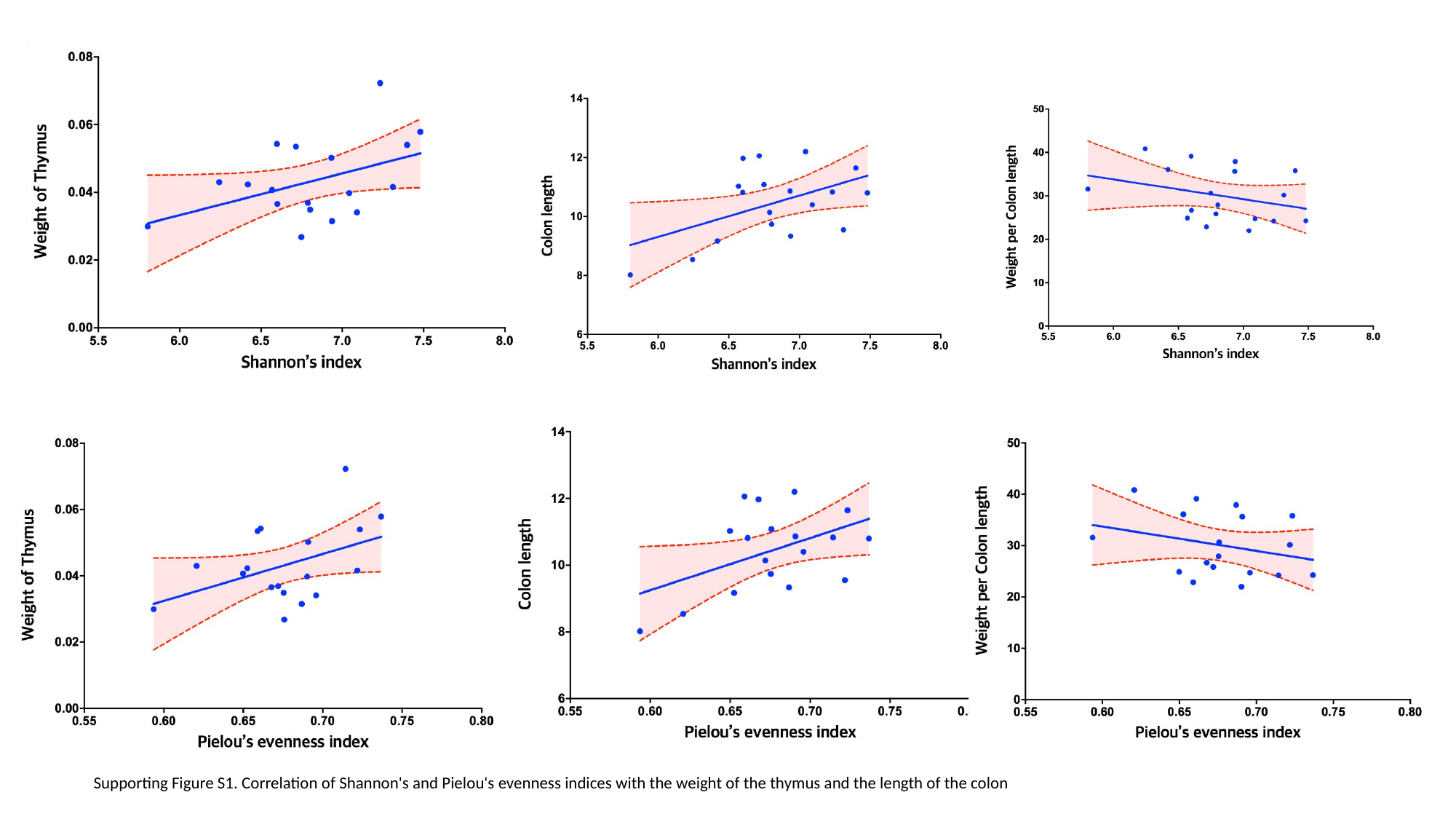

Supporting Figure S1. Correlation of Shannon's and Pielou's evenness indices with the weight of the thymus and the length of the colon

## Slide 3
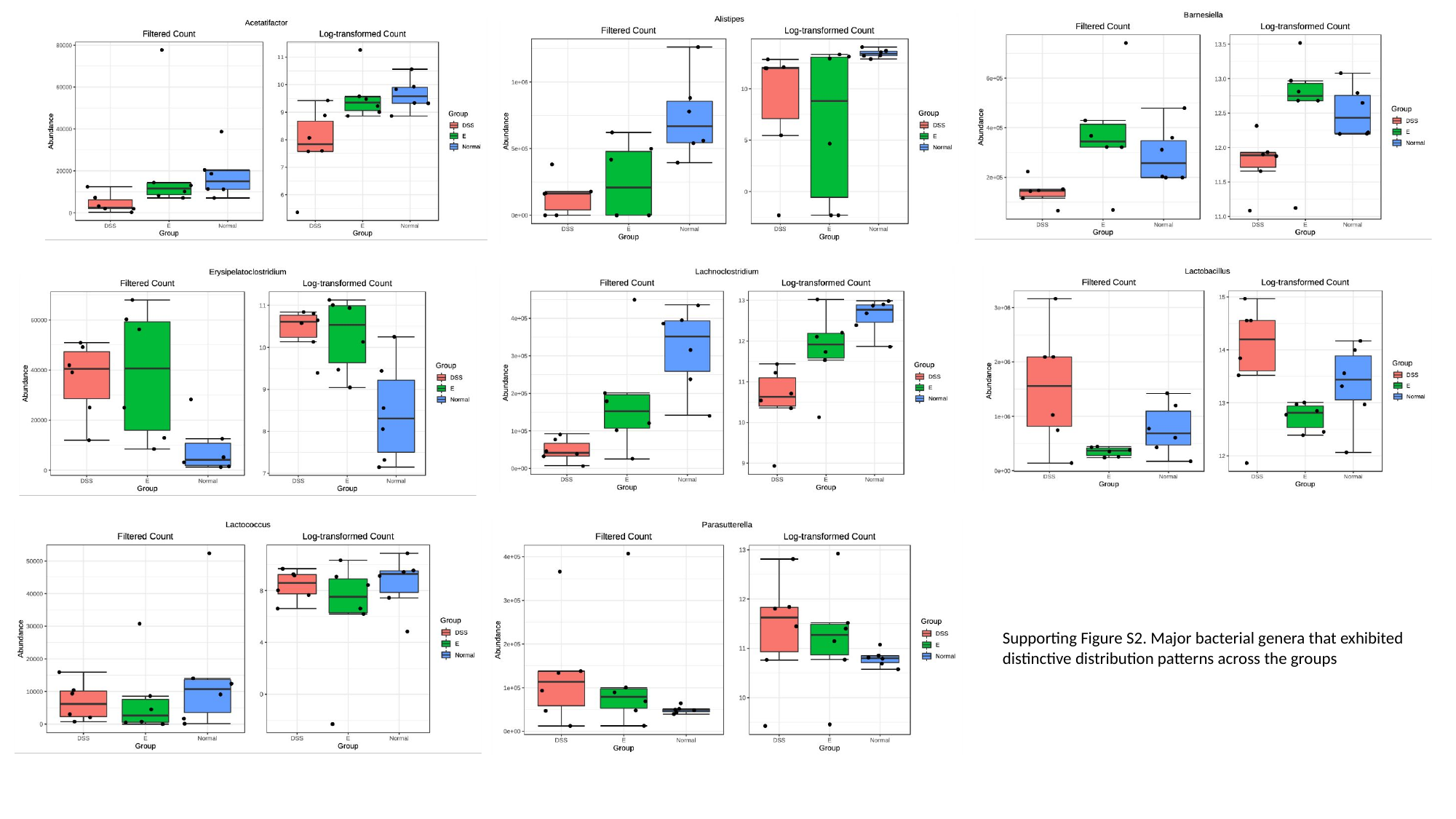

Supporting Figure S2. Major bacterial genera that exhibited distinctive distribution patterns across the groups

## Slide 4
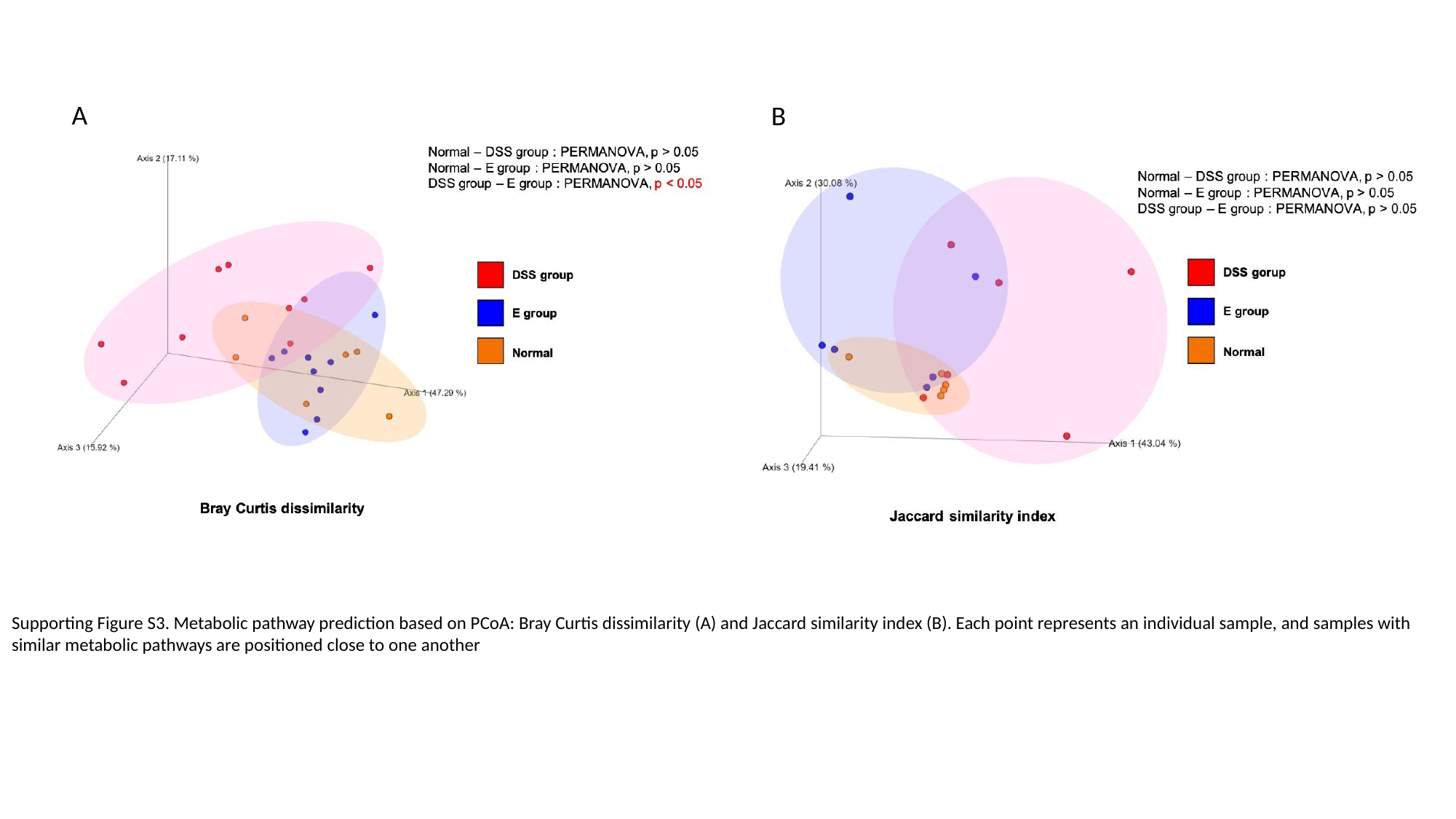

A
B
Supporting Figure S3. Metabolic pathway prediction based on PCoA: Bray Curtis dissimilarity (A) and Jaccard similarity index (B). Each point represents an individual sample, and samples with similar metabolic pathways are positioned close to one another
