# Supplementary material for: Ameliorative Effects of Newly Developed Citrus Hybrid “Mubong” Peel Extract on Experimental Colitis and Gut Microbiota Dysbiosis
Source: Food Sci Nutr. 2026 Jul 9;14(7):e72098. doi: 10.1002/fsn3.72098 (PMC13349115; doi:10.1002/fsn3.72098)
Supplement: Supplementary file 2 — Table S1: The composition of volatile organic compounds of Mubong peel and flesh extracts; a = alcohols; b = aldehydes; c = esters; d = hydrocarbons; e = ketones; f = etc. [file FSN3-14-e72098-s002.docx]

**Ameliorative Effects of Newly Developed Citrus Hybrid ‘Mubong’ Peel Extract on Experimental Colitis and Gut Microbiota Dysbiosis**

**Awraris Derbie Assefa1†, Sang Suk Kim2†, Seung-Gab Han1, YoSup Park1, Jee-Soo Park1***

^1^Citrus Research Center, National Institute of Horticultural & Herbal Science, Rural Development Administration, Seogwipo 63607, Korea, Republic of

^2^Department of Herbal Crop Research, National Institute of Horticultural & Herbal Science, Rural Development Administration, Eumseong 27709, Korea, Republic of

^3^Department of Horticultural Science, School of Horticulture and Forest, Mokpo National University, Muan 58554, Korea, Republic of.

*Correspondence: Jee-Soo Park, Email: jeespark@korea.kr

† These authors should be considered joint first author

Supporting Table S1. The composition of volatile organic compounds of Mubong peel and flesh extracts; a=alcohols; b=aldehydes; c=esters; d=hydrocarbons; e=ketones; f =etc

| NO. | RT | Compound | Similarity index | Peel | Flesh |
| --- | --- | --- | --- | --- | --- |
| 1 | 7.667 | α-Tujene ^d^ | 94 | 0.451 | - |
| 2 | 7.839 | α-Pinene ^d^ | 96 | 0.737 | 0.682 |
| 3 | 9.275 | Sabinene^d^ | 97 | 2.227 |  |
| 4 | 10.036 | β-Myrcene^d^ | 94 | 3.883 | 2.761 |
| 5 | 10.866 | α-Terpinene^d^ | 98 | 0.193 | 0.161 |
| 6 | 11.089 | o-Cymene^d^ | 94 | - | 0.174 |
| 7 | 11.696 | d-Limonene^d^ | 89 | 69.059 | 88.713 |
| 8 | 11.827 | cis-Ocimene^d^ | 96 | 0.277 | 0.111 |
| 9 | 12.188 | β-Ocimene^d^ | 98 | 3.061 | 1.218 |
| 10 | 12.457 | γ-Terpinene^d^ | 97 | 0.377 | 0.733 |
| 11 | 12.708 | (Z)-Sabinene hydrate^d^ | 91 | 0.104 | - |
| 12 | 13.509 | α-Terpinolene^d^ | 98 | 0.208 | 0.212 |
| 13 | 14.116 | Linalool^a^ | 97 | 7.818 | 0.288 |
| 14 | 14.185 | Nonanal^b^ | 90 | 0.098 | 0.136 |
| 15 | 15.861 | Citronellal^b^ | 98 | 0.062 | - |
| 16 | 16.410 | 4-Terpinenol^a^ | 97 | - | 0.32 |
| 17 | 16.668 | (E)-p-Mentha-2,8-dienol^a^ | 93 | 0.304 | - |
| 18 | 16.725 | α-Terpineol^a^ | 91 | 0.152 | - |
| 19 | 16.839 | cis-Dihydrocarvone^e^ | 99 | 0.328 | - |
| 20 | 16.983 | trans-Dihydrocarvone^e^ | 98 | 0.115 | - |
| 21 | 17.063 | Decanal^b^ | 91 | 0.573 | - |
| 22 | 17.126 | α-Methyl cinnamic aldehyde^b^ | 91 | 0.413 | - |
| 23 | 17.263 | cis-Carveol^a^ | 98 | 0.313 | - |
| 24 | 17.458 | p-Mentha-1,8-dien-6-ol^a^ | 98 | 0.383 | - |
| 25 | 17.652 | Carvone^e^ | 96 | 2.158 | - |
| 26 | 18.058 | Perilla aldehyde^b^ | 96 | 0.430 | - |
| 27 | 18.373 | Perilla alcohol^a^ | 95 | 0.119 | - |
| 28 | 18.756 | Dodecamethylcyclohexasiloxane^f^ | 94 | - | 0.109 |
| 29 | 19.065 | Carvyl acetate E^e^ | 94 | 0.695 | - |
| 30 | 19.243 | Geranyl acetate^e^ | 95 | 0.126 | - |
| 31 | 19.397 | Tetradecane^d^ | 98 | 0.129 | - |
| 32 | 19.632 | β-Caryophyllene^d^ | 99 | 0.167 | - |
| 33 | 19.712 | Perillyl acetate^d^ | 98 | 0.174 | - |
| 34 | 19.838 | Geranyl acetone^e^ | 90 | - | 0.221 |
| 35 | 19.918 | Humulene^d^ | 90 | 0.079 | - |
| 36 | 20.136 | Germacrene-D^d^ | 99 | 0.089 | - |
| 37 | 20.273 | α-Farnesene^d^ | 96 | - | 0.184 |
| 38 | 20.433 | δ-Cadinene^d^ | 99 | 0.055 | - |
| 39 | 20.668 | Nerolidol^a^ | 93 | 0.036 | - |
| 40 | 22.316 | Nootkatone^a^ | 99 | 0.056 | - |
|  |  | Total |  | 95.449 | 96.023 |
